# Porous vanadium dioxide thin film-based Fabry−Perot cavity system for radiative cooling regulating thermochromic windows: experimental and simulation studies

**DOI:** 10.1515/nanoph-2023-0716

**Published:** 2024-01-18

**Authors:** Saranya Bhupathi, Shancheng Wang, Guanya Wang, Yi Long

**Affiliations:** School of Materials Science and Engineering, Nanyang Technological University, 50 Nanyang Avenue, 639798, Singapore, Singapore; Singapore-HUJ Alliance for Research and Enterprise (SHARE), Campus for Research Excellence and Technological Enterprise (CREATE), 138602, Singapore, Singapore; Department of Electronic Engineering, The Chinese University of Hong Kong, Shatin, New Territories, Hong Kong SAR 999077, China

**Keywords:** radiative cooling, thermochromic window, Fabry−Perot resonator, sculptured thin film, porous VO_2_

## Abstract

Radiative cooling in smart windows using VO_2_ – a dynamic thermal management material, is of potential interest for enhancing energy savings in buildings due to its both solar and emittance tuneability in response to changing temperatures. However, studies related to the effects of VO_2_ thin film microstructure in a multilayer system on emissivity regulation are currently lacking. The present study addresses the thermochromic and emissivity performance of VO_2_/ZnSe/ITO/Glass Fabry−Perot (F–P) cavity thin film system, by manipulating the porosity in VO_2_ thin film. The device is fabricated by commercially feasible physical vapor deposition methods such as sputtering and thermal evaporation, most suitable for mass production. The optimized sample with porous VO_2_ delivers an enhanced long-wave infrared (LWIR) emissivity contrast of Δ*ɛ*_LWIR_ ≥ 0.4 preserving a high visible transparency *T*_lum(avg)_ of ∼41 % compared to dense VO_2_. Then finite difference time domain (FDTD) simulation is performed to further understand the effects of varying VO_2_ porosity and ZnSe thickness on the F–P cavity properties. The reduced low-temperature *ɛ*_LWIR_ (0.1–0.2) gives this film better energy saving in regions where warming demand is dominant as simulated by EnergyPlus.

## 1 Introduction

Cooling of buildings to create a pleasant living environment is in great demand as an outcome of global warming, rapid population expansion, and mass migration to urban areas. According to the latest report by the International Energy Agency (IEA), the operational energy demands in buildings for indoor heating, cooling, lighting, etc. increased by 4 % from 2020, with the energy demand for space cooling particularly raised to >5 % from 2021 [1], [2]. Huge investments are being made in improving the energy performance of existing buildings and transitioning towards sustainable buildings to significantly minimize energy consumption and CO_2_ emissions. Passive cooling by radiation is a potential alternative to conventional active cooling methods for thermal comfort in buildings by emitting thermal radiation into outer space through a long-wave infrared (LWIR) atmospheric window (8–14 µm) with reduced energy consumption and addressing the environmental impacts. In dynamic radiative cooling technique, the LWIR emissivity can be tuned and regulated with the ambient temperature using a smart material. The ability to respond to varying environmental conditions makes dynamic radiative cooling a robust approach, offering seasonal applicability and regional adaptability in diverse geographical and climatic regions [3], [4]. Thermochromic smart material [5] is an excellent choice for dynamic radiative cooling operation, as it can function as a self-adaptive smart window by integrating thermochromic and radiative cooling concepts.

Vanadium dioxide (VO_2_) is a widely studied thermochromic material that undergoes a reversible phase transition from a low-temperature semiconducting monoclinic phase (VO_2_-M) into a high-temperature metallic tetragonal (rutile) phase (VO_2_-R) at a transition temperature of (*T*_*c*_) 68 °C [6]–[8]. Due to its near room temperature (RT) phase transition, ability to modulate solar transmittance, and tunable thermal emissivity in the LWIR range, VO_2_ emerges as a promising candidate to realize energy-efficient radiative cooling regulating thermochromic (RCRT) smart windows. The emissivity of VO_2_ can be dynamically tuned (Δ*ɛ* = *ɛ*_hot_ − *ɛ*_cold_) upon temperature by proper selection of substrate, coating material, and multilayer structure. VO_2_ deposited on a metal substrate (e.g. Al) shows positive emissivity-switching (Δ*ɛ* > 0) [9], while VO_2_ on glass, sapphire, and quartz substrates exhibits negative emissivity-switching (Δ*ɛ* < 0) [10]. For an RCRT window, it is desirable to have a positive emissivity difference i.e., a low thermal emissivity (*ɛ*_cold_) in colder ambient to maintain indoor heat and high thermal emissivity (*ɛ*_hot_) in hotter ambient to release the excess heat.

The thermochromic smart window based on VO_2_ is a widely investigated topic of research that is aimed at regulating solar radiation while maintaining high luminous transmittance with changing seasons. The studies focusing on RCRT windows using VO_2_ have started to emerge in recent years. Various VO_2_-based designs have been studied to smartly regulate the window’s emissivity. A low emissivity thermochromic smart window was achieved using double-layered films of ZnO:Al/VO_2_ and Pt/VO_2_ on a silica substrate [11], [12]. A low and static emissivity at both hot and cold seasons was realized in these works to improve the window’s thermal insulating ability and reduce energy exchange. Dynamic emissivity control has been demonstrated for the thermal management of spacecraft by incorporating VO_2_-based multilayer switchable radiator designs [13]–[15]. Similar multilayer designs have been adapted in thermochromic smart windows implementing Fabry−Perot (F–P) cavity, photonic crystal, and bio-inspired optical designs [16]–[18]. S. Wang et al. designed a VO_2_/PMMA/ITO/Glass/ITO F–P transparent multilayer, delivering an enhanced Δ*ɛ* of 0.4, attaining 0.21 at cold and 0.61 at hot states [4]. Thermal emissivity contrast of 0.26 (0.8 at hot and 0.54 at cold) was obtained in the VO_2_ metasurface combined with F–P structure (VO_2_/SiO_2_/AZO/CaF_2_) by exploiting the plasmonic enhancement effects [19]. Besides VO_2_, other smart materials have been studied in thermochromic windows possessing positive emissivity switching. A solar and thermal regulatory window made of poly(N-isopropylacrylamide)-silver nanowire composite film delivered an emittance modulation of 0.57 with enhanced thermochromic performance [20]. LWIR modulation ability of 0.5 was attained in solar/RC dual-control smart window by integrating a hydrogel composite with polydimethylsiloxane-based kirigami structure for emissivity tuning [21]. In the actual applications of smart windows, besides high luminous transmittance *T*_lum_, various regions require optimized solar transmission and emission. For example, in climate zones 5, 6, and 7, low emission and high solar transmission are favored [4]. A facile way is needed to offer complicated tunability in various regions in the smart window. More microstructure-performance relationship studies could offer a platform for optical design in specific regions. Notably, the microstructure and its influence on the emissivity are ambiguous in the literature that used thermochromic smart materials in the sample system [19], [22]–[25]. The VO_2_ microstructure modification approach via porosity control could be interesting to understand and upgrade the RCRT window’s performance for specific zones.

In this work, we demonstrate the thermochromic performance and emittance switching of VO_2_/ZnSe/ITO/Glass F–P cavity multilayer system by modifying VO_2_ microstructure into porous. We adopt industrially desirable sputtering technique to study the porosity effects on the *T*_lum_/*T*_sol_/Δ*ɛ*_LWIR_ manipulation. The optical studies are performed in the thin film samples with porous and dense VO_2_ microstructures, modifying the ZnSe spacer layer with thicknesses of 75 and 150 nm. An enhanced positive emissivity modulation (Δ*ɛ*_LWIR_) of ≥ 0.4, together with high luminous transmittance (40.9 %) and much-reduced low-temperature emissivity (*ɛ*_LWIR(cold)_) is achieved in the system with porous VO_2_ and the results are correlated with the VO_2_ microstructure. We compare the obtained results with those from dense VO_2_ thin films. We also present the transmittance and emittance switching characteristics obtained from the finite difference time domain (FDTD) simulation. Finally, energy-saving performance from building energy consumption (EnergyPlus) simulation using porous and dense VO_2_ is compared and the applicability in specific climate zones is discussed.

## 2 Materials and methods

### 2.1 F–P cavity design and operation

**Figure 1:**
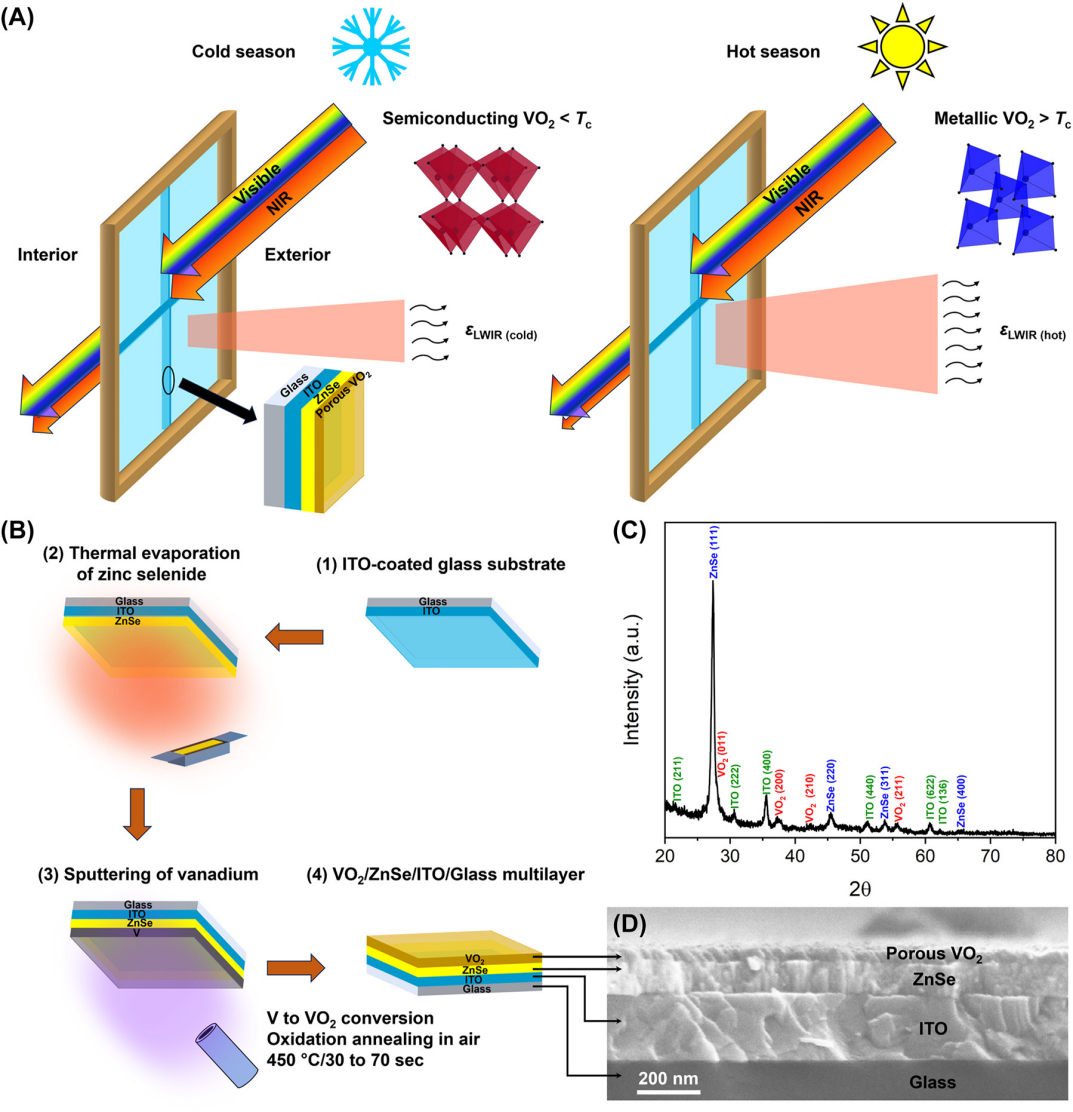
RCRT window concept, VO_2_/ZnSe layer fabrication steps, and microstructural characterization. (A) 3D schematic demonstrates the operation of VO_2_/ZnSe/ITO/Glass RCRT windowpane. In cold season, below the *T*_*c*_ of VO_2_, the reflective nature of window to LWIR delivers low emissivity (*ɛ*_LWIR(cold)_) maintaining thermal comfort in the interior. In hot season, above the *T*_*c*_ of VO_2_, the absorptive nature of window to LWIR delivers high emissivity (*ɛ*_LWIR(hot)_) releasing surplus heat from the interior. High luminous transmittance and lower heat gain are maintained in both cases. (B) Schematic diagram of VO_2_/ZnSe layer fabrication on the ITO-coated glass substrate by sputtering and thermal evaporation. (C) GIXRD pattern of porous VO_2_/ZnSe/ITO/Glass multilayer annealed at 450 °C for 2 min. (D) SEM cross-section of porous VO_2_ obtained in the OAD configuration at *α* = 85° deposition angle. The SEM image corresponds to sample S4 annealed at 450 °C for 50 s in the ambient air.

Figure 1A depicts the 3D schematic of the RCRT window made of VO_2_/ZnSe/ITO/Glass multilayer and its operation in cold and hot seasons. The device consists of a ZnSe dielectric spacer layer sandwiched between the top VO_2_ layer and the bottom low-E ITO layer constructed on a glass substrate. The VO_2_/ZnSe/ITO multilayer system acts like an F–P cavity. ZnSe is chosen as a dielectric spacer layer because of its characteristics such as high transparency in visible and atmospheric window wavelength as well as low optical absorption [26]. The ZnSe F–P cavity involves in the resonation of the LWIR region which helps to enhance the LWIR emissivity switching (Δ*ɛ*) between cold and hot states. Below the phase transition of VO_2_ (*T* < *T*_*c*_), the high transparency of VO_2_ and ZnSe spacer to LWIR facilitates the RCRT system to be highly reflective in the LWIR region. It leads to a low emissivity in the cold state (*ɛ*_LWIR(cold)_). VO_2_ becomes metallic above the phase transition (*T* > *T*_*c*_) which functions as a top mirror in the F–P cavity resonator. It leads to an increased emissivity at hot state (*ɛ*_LWIR(hot)_) due to enhanced absorption amplifying the radiative cooling. In both seasons, emissivity is regulated according to the varying exterior temperature, maintaining a high visible transmittance, and lowering the heat gain in the interior.

### 2.2 Fabrication of porous and dense VO_2_ thin films

The thin film multilayer samples are prepared using Lesker PVD 75 Deposition System facilitated with thermal evaporation and sputtering. ZnSe thin films are fabricated by thermal evaporation using tantalum as an evaporation boat source. The porous and dense VO_2_ thin films are fabricated by sputtering with a vanadium (V) target of purity = 99.9 %, diameter = 50 mm, thickness = 6.3 mm from Kurt J. Lesker. ITO-coated glass substrates of 20 × 20 mm^2^ are used as thin film substrates. The deposition is performed at a base pressure of 5 × 10^−5^ Torr. The pre-sputtering is performed for 10 min before each deposition by keeping the shutter closed, to eliminate any oxide contamination on the V target. The porous and dense V thin films are deposited in pure argon at a pressure of 2 and 17 mTorr, respectively, at a DC power of 80 W. A regular hot plate (IKA C-Mag HS 7) is used to oxidize V into VO_2_.

Figure 1B depicts the sequential steps involved in the fabrication of the VO_2_/ZnSe/ITO/Glass RCRT multilayer system. ITO-coated glass substrates are first ultrasonically cleaned in deionized (DI) water and ethanol separately for 10 min before the ZnSe deposition. Then the 75 and 150 nm thicknesses of ZnSe individual spacer layers are deposited by thermal evaporation as a second step. The ZnSe growth rate during thermal evaporation is monitored by a quartz crystal microbalance (QCM). In the third step, a top V layer of 35 nm thickness is fabricated by sputtering with V target sputtered at RT. In normal deposition configuration, the flux direction normal to the substrate generates thin film of vertical nanocolumn. In oblique angle deposition (OAD) configuration, the arrival of vapor flux in oblique angle results in the porous thin film with tilted nanocolumn. The film microstructure and porosity can be tailored by incident flux angle and substrate rotation in the OAD [27], [28]. In this work, the normal configuration is used to attain flat VO_2_ thin films with dense microstructure, while the OAD configuration is used to attain porous VO_2_ thin films. A custom-designed wedge structure with a 5° apex angle is used to perform the OAD at a deposition angle *α* = 85°. The fabrication details of VO_2_ in OAD configuration and the systematic microstructural investigation are elaborated in our previous work [29]. In the final step, the as-prepared V thin film samples are transformed into VO_2_ by oxidation annealing in the ambient air at 450 °C by changing the annealing time from 30 to 70 s in steps of 10 s.

### 2.3 Microstructural characterization

Thin film crystalline phases are studied by grazing incidence X-ray diffraction (GIXRD) using the Shimadzu XRD-6000 system, equipped with CuKα radiation of *λ* = 1.5418 Å . The measurements are performed at 0.5° fixed theta at a scanning speed of 0.4° min^−1^. The thin film surface morphology, and thickness are characterized by a field emission scanning electron microscope (FESEM, SUPRA 55, Carl Zeiss) at an accelerating voltage of 2–8 keV. The thin film sample roughness is examined by an atomic force microscope (AFM, DI-3100, Bruker, Germany) using a silicon cantilever in tapping mode at a scanning frequency of 1 Hz.

### 2.4 Optical characterization

The temperature-controlled transmittance spectrum is recorded using a UV–Vis–NIR spectrophotometer from AvaSpec spectrometers, Avantes, Netherlands, equipped with a heating and cooling stage (T95-PE, Linkam, U.K.) to determine the transmittance parameters such as *T*_lum_, *T*_NIR_, *T*_sol_, Δ*T*_lum_, Δ*T*_NIR_, and Δ*T*_sol_. The visible, near-infrared (NIR), and solar energy transmittance of a thermochromic smart window can be determined from the integration of luminous transmittance (*T*_lum_, 380–780 nm), NIR transmittance (*T*_NIR_, 780–2500 nm), and solar transmittance (*T*_sol_, 300–2500 nm), respectively, which can be expressed as follows:

(1) $$T_{\text{lum}}=\frac{\overset{780}{\underset{\lambda =380}{\int }}\varphi_{\text{lum}}T\left(\lambda \right)\text{d}\lambda }{\overset{780}{\underset{\lambda =380}{\int }}\varphi_{\text{lum}}\text{d}\lambda }$$

(2) $$T_{\text{NIR}}=\frac{\overset{2500}{\underset{\lambda =780}{\int }}AM_{1.5}\left(\lambda \right)T\left(\lambda \right)\text{d}\lambda }{\overset{2500}{\underset{\lambda =780}{\int }}AM_{1.5}\left(\lambda \right)\text{d}\lambda }$$

(3) $$T_{\text{sol}}=\frac{\overset{2500}{\underset{\lambda =300}{\int }}AM_{1.5}\left(\lambda \right)T\left(\lambda \right)\text{d}\lambda }{\overset{2500}{\underset{\lambda =300}{\int }}AM_{1.5}\left(\lambda \right)\text{d}\lambda }$$

where *T*(*λ*) represents measured spectral transmittance, *φ*_lum_ is the photopic luminous efficiency of the human eye [30], and AM_1.5_ represents air mass 1.5 solar irradiance spectrum distributions (corresponding to the sun standing 37° above the horizon) [31].

The luminous, NIR, and solar transmittance modulations at cold (20 °C) and hot (100 °C) temperatures can be represented as follows:

(4) $$\Delta T_{\text{lum}}=T_{\text{lum}\left(\text{cold}\right)}-T_{\text{lum}\left(\text{hot}\right)}$$

(5) $$\Delta T_{\text{NIR}}=T_{\text{NIR}\left(\text{cold}\right)}-T_{\text{NIR}\left(\text{hot}\right)}$$

(6) $$\Delta T_{\text{sol}}=T_{\text{sol}\left(\text{cold}\right)}-T_{\text{sol}\left(\text{hot}\right)}$$

where Δ*T*_lum_, Δ*T*_NIR_, and Δ*T*_sol_ indicate the luminous, NIR, and solar modulation abilities, respectively.

The integrated LWIR emissivity is estimated with a dual-band emissivity measuring equipment (IR-2, Shanghai Chengbo Photoelectric Technology) with a heating stage. The emissivity values are measured at five different spots in one sample, and the values are averaged to estimate the final integrated LWIR emissivity (*ɛ*_LWIR_). The emissivity difference (Δ*ɛ*_LWIR_) is calculated from the following equation:

(7) $$\Delta \varepsilon_{\text{LWIR}}=\varepsilon_{\text{LWIR}\left(\text{hot}\right)}-\varepsilon_{\text{LWIR}\left(\text{cold}\right)}$$

where *ɛ*_LWIR(hot)_ and *ɛ*_LWIR(cold)_ are the integrated LWIR emissivity at hot and cold temperatures, respectively.

The Fourier transform infrared (FTIR) spectra are collected with an FTIR spectrometer (Perkin Elmer Frontier) equipped with an integrating sphere, in the wavelength ranging from 2.5 to 25 μm. The emissivity curve *ɛ*(*λ*) is plotted according to Kirchhoff’s law of thermal radiation: *ɛ*(*λ*) = *A*(*λ*) = 1 − *T*(*λ*) − *R*(*λ*). The spectral absorptivity *A*(*λ*) and emissivity *ɛ*(*λ*) are equal for an object at thermal equilibrium. *R*(*λ*) and *T*(*λ*) are the spectral reflectance and transmittance measured by the FTIR spectrometer.

### 2.5 FDTD simulation

In FDTD simulation, a stacked VO_2_/ZnSe/ITO/SiO_2_ multilayer structure is constructed for analysis. The VO_2_ layer is designed as a non-compact array of titled micropillars to approximate the porous structure, and another non-porous VO_2_ layer featuring a dense structure composed of a compact array is designed for comparison. The refractive index of VO_2_ at cold and hot states [32], ZnSe [33], ITO [34], and SiO_2_ [35] are obtained from the previous reports. The initial thickness of VO_2_ and ZnSe is set to 30 nm and 1200 nm respectively. Periodic, symmetric, and perfectly matched layers (PML) are set as the boundary conditions in the *x*-, *y*-, and *z*-direction of the model during the simulation. A normal incident plane wave is built above the multilayer to simulate the solar source. Besides, two monitors are set up above the light source and below the multilayer to obtain reflectance and transmittance spectra. All the simulation results are calculated with the mesh size of 0.01 × 0.01 × 0.01 μm^3^.

### 2.6 EnergyPlus simulation

The EnergyPlus is used in actual building energy consumption simulation. In the simulation, a building model with the dimensions of 8 m in length, 6 m in width, and 2.7 m in height is used. The floor area is 48 m^2^ and the total external wall surface area of the building is 75.6 m^2^. Four windows with the dimensions of 3 m in width and 2 m in height are installed in the four orientations to avoid the impact of orientation. The windows cover 31.7 % of the total wall surface area. The structure of the model house is shown in Figure S1. Hourly weather data for a Typical Meteorological Year (TMY) are employed as the external boundary conditions. The weather data of Whitehorse, Moscow, Berlin, Melbourne, Cairo, and Singapore are used in this simulation [36]. The energy usage in the unit of MJ/m^2^ is calculated based on the floor area.

## 3 Results and discussion

### 3.1 Microstructural analysis

The XRD pattern of porous VO_2_ with 100 nm thickness on ZnSe/ITO/Glass multilayer annealed at 450 °C for 2 min is shown in Figure 1C. The XRD pattern comprises peaks from VO_2_, ZnSe, and ITO layers. The peak positions of ZnSe at 27.4°, 45.5°, 53.8°, and 65.3° correspond to reflections from (111), (220), (311), and (400) planes, respectively. The obtained diffraction peaks are indexed to the cubic structure of *F*43*m* space group matching with the Match database no 00-005-0522. The peaks observed for ITO at 21.6°, 30.6°, 35.5°, 51.1°, 60.7°, and 62.4° associated with reflections from (211), (222), (400), (440), (622), and (136) planes, respectively, crystallizing in cubic structure with *Ia*3 space group (Match database no: 01-089-4597). VO_2_ exhibits peaks at 28.0°, 37.4°, 42.4°, and 55.7° are reflections from (011), (200), (210), and (211) planes, respectively. The diffraction peaks of VO_2_ confirm the formation of monoclinic VO_2_(M) with the space group *P*21/*c* which is in very good accordance with Match database no: 00-009-0142. The average crystallite size of VO_2_ estimated from XRD using the Scherrer formula is about 30 nm. XRD confirms the formation of expected crystalline phases of monoclinic VO_2_ and ZnSe thin films on the ITO-coated glass substrate.

**Figure 2:**
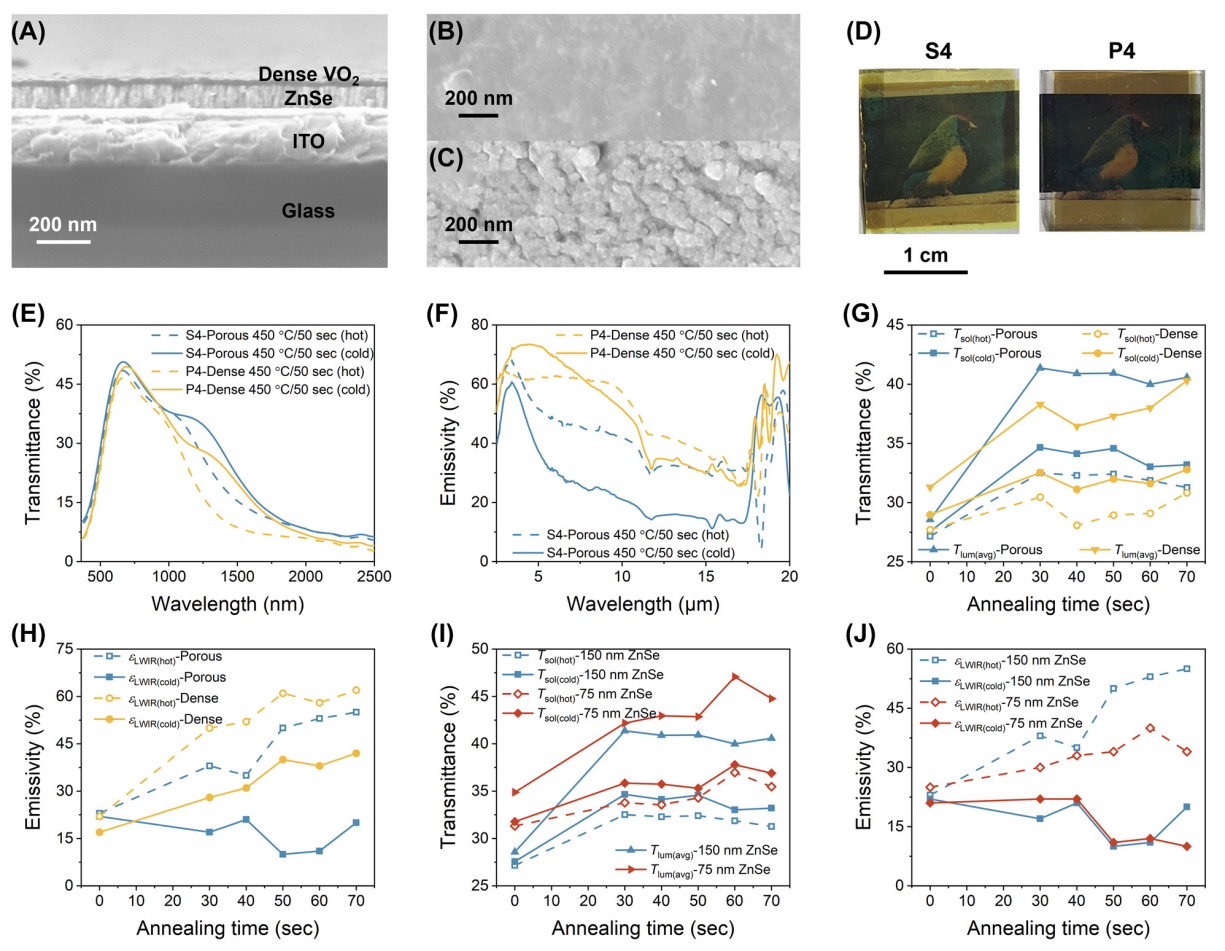
Microstructural characterization and optical performance of VO_2_/ZnSe/ITO/Glass samples. (A) SEM cross-section of sample P4 with dense VO_2_ obtained in the normal configuration. SEM top-view images of (B) sample P4 dense VO_2_ and (C) sample S4 porous VO_2_. (D) Images of porous and dense VO_2_ samples S4 and P4 respectively, heat treated at 450 °C for 50 s. (E) Comparison of transmittance spectra between porous (S4) and dense (P4) VO_2_ in the Vis-NIR range at cold and hot states. (F) Comparison of emissivity spectra between porous (S4) and dense (P4) VO_2_ in the LWIR range at cold and hot states. Comparison of (G) *T*_lum(avg)_, *T*_sol_ at cold/hot states and (H) *ɛ*_LWIR_ at cold/hot states between samples of porous (S1–S6) and dense (P1–P6) microstructures versus annealing time. Comparison of (I) *T*_lum(avg)_, *T*_sol_ at cold/hot states and (J) *ɛ*_LWIR_ at cold/hot states between porous samples of different spacer thicknesses such as 150 nm (S1–S6) and 75 nm (S11–S16) versus annealing time. Sample S1 corresponds to as-prepared porous V(35 nm)/ZnSe(150 nm) without heat treatment. Samples S2–S6 correspond to porous VO_2_(35 nm)/ZnSe(150 nm), heat treated at 450 °C for 30, 40, 50, 60, and 70 s, respectively. Sample P1 corresponds to as-prepared dense V(35 nm)/ZnSe(150 nm) without heat treatment. Samples P2–P6 correspond to dense VO_2_(35 nm)/ZnSe(150 nm) heat treated at 450 °C for 30, 40, 50, 60, and 70 s, respectively. Sample S11 corresponds to as-prepared porous V(35 nm)/ZnSe(75 nm) without heat treatment. Samples S12–S16 correspond to porous VO_2_(35 nm)/ZnSe(75 nm), heat treated at 450 °C for 30, 40, 50, 60, and 70 s, respectively.

The SEM cross-section pictures of porous and dense VO_2_ annealed at 450 °C for 50 s are given in Figures 1D and 2A, respectively. The SEM picture depicts the arrangement of the VO_2_/ZnSe stack on the ITO-coated glass substrate, with a clear interface that can be observed between each layer. The VO_2_ deposited in the OAD configuration exhibits a tilted columnar structure throughout the whole thickness with a column tilt angle of 30°. The columnar tilting and the porosity arise from the shadowing effect due to the oblique incidence (>85°) and limited adatom diffusion because of the ballistic movement of the vapor flux [37]. The boundary between tilted nanocolumns can be seen in Figure 1D, which is used to estimate the columnar diameter and the value falls in the range of 15–35 nm. Whereas the nanocolumns of VO_2_ prepared in normal configuration seem closely packed in SEM cross-section (Figure 2A) resulting in a non-porous and dense surface morphology without cracks (Figure 2B). Whereas the surface morphology of VO_2_ that emerged from OAD has circular grains that look porous and crack-free (Figure 2C) which extend throughout the film surface. The surface roughness estimated from AFM for porous VO_2_ is 4 nm (Figure S2A and B), while for dense VO_2_ it is 2.3 nm (Figure S2C and D), which shows that the porous VO_2_ surface is slightly rougher than the dense VO_2_. Therefore, SEM and AFM studies confirm the microstructure of VO_2_ is porous for samples attained in OAD configuration, while it is non-porous and dense for normal configuration. Figure 2D represents the images of porous and dense VO_2_ samples S4 and P4 respectively, annealed at 450 °C for 50 s taken with a background picture. It shows that both the samples are transparent. However, the dense VO_2_ is slightly darker than the porous sample which indicates that the porous VO_2_ is more transparent than the dense VO_2_.

### 3.2 Optical characterization

For transmittance and emissivity measurements, porous and dense VO_2_ samples with changing spacer thicknesses of 150 and 75 nm are fabricated. As-prepared samples without annealing such as porous V(35 nm)/ZnSe(150 nm)/ITO/Glass (sample S1) and porous V(35 nm)/ZnSe(75 nm)/ITO/Glass (sample S11) are prepared in the OAD configuration, while dense V(35 nm)/ZnSe(150 nm)/ITO/Glass (sample P1) and dense V(35 nm)/ZnSe(75 nm)/ITO/Glass (sample P11) are prepared in the normal configuration. Each of the as-prepared samples has been annealed at 450 °C for 30–70 s in steps of 10 s in the ambient air. Transmittance and emissivity are studied in both as-prepared and annealed, porous, and dense VO_2_ samples to check the influence of microstructure and spacer thickness effects on thermochromic and emissivity parameters. All the transmittance and emissivity spectra are acquired at both cold and hot states.

Samples S2–S6 correspond to porous VO_2_(35 nm)/ZnSe(150 nm)/ITO/Glass annealed at 450 °C for 30–70 s, respectively. Samples P2–P6 correspond to dense VO_2_(35 nm)/ZnSe(150 nm)/ITO/Glass annealed at 450 °C for 30–70 s, respectively. Samples S12–S16 correspond to porous VO_2_ with a spacer thickness of 75 nm annealed at 450 °C for 30–70 s, respectively. Similarly, samples P12–P16 correspond to dense VO_2_ with 75 nm ZnSe spacer annealed at 450 °C for 30–70 s respectively. The thermochromic and emissivity parameters of porous S1–S6 and dense P1–P6 are summarized in Table 1. The thermochromic and emissivity parameters of porous S11–S16 and dense P11–P16 are summarized in Table S1.

**Table 1:** Summary of experimental thermochromic and emissivity parameters of porous and dense V and VO_2_ samples with sample codes S1–S6 and P1–P6. The annealing temperature of samples S2–S6 and P2–P6 is 450 °C.

Code	Layer and thickness details	Annealing	*T*_lum_ (%)		*T*_lum(avg)_ (%)	Δ*T*_NIR_ (%)	*T*_sol_ (%)		Δ*T*_sol_ (%)	*ɛ*_LWIR_		Δ*ɛ*_LWIR_
		time (s)	20 °C	100 °C			20 °C	100 °C		20 °C	100 °C	
S1	Porous V(35 nm)/ZnSe(150 nm)	As-prepared	29.0	28.2	28.6	0.0	27.6	27.2	0.4	0.22	0.23	0.01
S2	Porous VO_2_(35 nm)/ZnSe(150 nm)	30	42.4	40.4	41.4	1.1	34.7	32.5	2.1	0.17	0.38	0.21
S3	Porous VO_2_(35 nm)/ZnSe(150 nm)	40	41.5	40.3	40.9	1.1	34.1	32.3	1.8	0.21	0.35	0.14
S4	Porous VO_2_(35 nm)/ZnSe(150 nm)	50	41.8	40.0	40.9	1.1	34.6	32.4	2.2	0.10	0.50	0.40
S5	Porous VO_2_(35 nm)/ZnSe(150 nm)	60	40.0	39.9	40.0	0.9	33.0	31.9	1.2	0.11	0.53	0.42
S6	Porous VO_2_(35 nm)/ZnSe(150 nm)	70	41.1	40.1	40.6	1.2	33.2	31.3	1.9	0.20	0.55	0.35
P1	Dense V(35 nm)/ZnSe(150 nm)	As-prepared	32.2	30.4	31.3	0.4	29.0	27.7	1.3	0.17	0.22	0.05
P2	Dense VO_2_(35 nm)/ZnSe(150 nm)	30	39.2	37.4	38.3	1.1	32.5	30.5	2.1	0.28	0.50	0.22
P3	Dense VO_2_(35 nm)/ZnSe(150 nm)	40	36.8	36.1	36.5	2.0	31.1	28.1	3.0	0.31	0.52	0.21
P4	Dense VO_2_(35 nm)/ZnSe(150 nm)	50	37.6	37.0	37.3	2.1	32.0	28.9	3.1	0.40	0.61	0.21
P5	Dense VO_2_(35 nm)/ZnSe(150 nm)	60	38.2	37.8	38.0	1.7	31.6	29.1	2.5	0.38	0.58	0.20
P6	Dense VO_2_(35 nm)/ZnSe(150 nm)	70	40.2	40.4	40.3	1.7	32.8	30.8	2.0	0.42	0.62	0.20

The transmittance and emissivity spectra at cold and hot states of porous S4 and dense P4 VO_2_ samples are given in Figure 2E and F, respectively. There are obvious changes observed in the transmittance and emissivity spectra of VO_2_ samples with porous and dense surface morphology. In Figure 2E, the transmittance spectra of the porous sample S4 in the visible and NIR regions are higher than that of the dense sample P4 at both cold and hot states. In Figure 2F, the emissivity spectrum of porous sample S4 in the cold state is lower than the emissivity of dense sample P4 in the cold state, and a similar trend is noticed in the hot state as well. The observed changes in the transmittance and emissivity spectra are attributed to the influence of the VO_2_ microstructural modification in the sample system. Further, all the annealed samples in porous and dense VO_2_ categories show positive emissivity-switching i.e., Δ*ɛ*_LWIR_ > 0 (Tables 1 and S1), a desired response for RC upon temperature variation. The lower emissivity values obtained in the cold state suggest the transparency of semiconducting VO_2_ and ZnSe layers to the LWIR region and the IR-reflective ITO [38], [39]. The higher emissivity at the hot state indicates LWIR absorption in the F–P cavity due to the VO_2_ phase transition into metallic. The emissivity switching is almost zero in all the as-prepared samples because of the absence of phase transition in the vanadium thin film.

To study the influence of microstructure, the *T*_lum(avg)_, *T*_sol_ at cold/hot states in Figure 2G and the emissivity at cold/hot states in Figure 2H between samples with porous (S1–S6) and dense (P1–P6) microstructures are compared, which are plotted versus the annealing time. Similarly, the *T*_lum(avg)_, *T*_sol_ at cold/hot states (left *y*-axis) and the emissivity at cold/hot states (right *y*-axis) between samples with porous (S11–S16) and dense (P11–P16) microstructures are compared and plotted versus the annealing time in Figure S3A.

The *T*_lum(avg)_ of porous VO_2_ samples from S2 to S6 in Figure 2G almost remained the same (∼40.8 %) with respect to the annealing time. A similar trend is observed for dense VO_2_ samples such as P2–P5 in Figure 2G and P12–P16 in Figure S3A. The *T*_lum(avg)_ of samples P2–P5 are almost ∼37.5 %, while for P12–P16 it is ∼40.2 %. A slight increment in *T*_lum(avg)_ of ∼6 % is noticed in the dense sample P6 (38 % for P5 to 40.3 % for P6) when the annealing time changes from 60 to 70 s. Whereas in porous VO_2_, the *T*_lum(avg)_ value of sample S14 with 50 s annealing time is ∼42.9 %, which increases to 47.1 % (∼10 % raise) when the annealing time changes to 60 s for sample S15. This value drops to 44.8 % for sample S16 with the annealing time of 70 s. An increase in *T*_lum(avg)_ at higher annealing time from 50 to 60 s can be correlated to the improved crystallinity of VO_2_ during oxidation annealing in the ambient air. The decrease in the *T*_lum(avg)_ at higher annealing time of 70 s may be due to the origination of V_*x*_O_*y*_ impurities [40], [41]. Moreover, 10 s step between each annealing time is inadequate to observe any substantial changes in the microstructure and chemical state of VO_2_ to correlate with the thermochromic parameters. Further investigation with longer annealing time is needed to validate this. The Δ*T*_sol_ values mainly emerge from the transmittance contrast in the NIR range [42], [43]. The Δ*T*_sol_ values obtained in porous and dense VO_2_ F–P systems are lower than the required optimal range of Δ*T*_sol_ > 10 % [44] which is due to the NIR-blocking nature of the ITO layer affecting the modulation abilities. It is also observed that the Δ*T*_sol_ values of dense VO_2_ are higher than the porous because of high coverage of VO_2_ [45]. However, the *T*_sol_ values of porous VO_2_ at cold and hot states are ∼1–22 % higher than the dense VO_2_.

Regarding the emissivity results between porous and dense VO_2_, the emissivity values of dense VO_2_ at cold and hot states are higher than the porous VO_2_ (Figures 2H and S3A). Specifically, the higher emissivity at cold state (*ɛ*_LWIR(cold)_ > 0.2–0.4) leads to a lower emissivity contrast (Δ*ɛ*_LWIR_) in all the dense VO_2_ samples. In the case of porous VO_2_, the *ɛ*_LWIR(cold)_ values are equivalent to the emissivity of ITO-coated glass substrate which is ∼0.1–0.2 (sample T1 in Table S2). The different resistive values of porous and dense VO_2_ are envisaged to alter the emissivity at cold and hot states [46]. Particularly, the Δ*ɛ*_LWIR_ values of porous VO_2_ annealed at 450 °C for 50, 60, and 70 s (samples S4–S6) are ≥ 0.4 which is relatively higher than the other porous and dense VO_2_ samples, and the values are comparable to our previously reported emissivity contrast value [4].

Considering the microstructure effect, overall the *T*_lum(avg)_ values of porous VO_2_ are comparatively higher than the dense VO_2_ as well as the optimal value (*T*_lum_ > 40 %) [44]. Furthermore, they are nearly 1.5 times higher than the *T*_lum(avg)_ value (∼27 %) reported in our previous work studied in VO_2_/PMMA/ITO/Glass/ITO system [4]. It is because the presence of air-filled nano-pores in porous VO_2_ originating from OAD contributes to the enhancement in *T*_lum_ through reducing refractive index and reflection loss [47], [48]. Also, higher *T*_sol_ and lower *ɛ*_LWIR_ at cold and hot states of VO_2_ samples with porous microstructure is beneficial to apply in climate zones 5, 6, and 7 for increasing natural daylighting and heating.

To study the effect of spacer thickness, the *T*_lum(avg)_, *T*_sol_ at cold/hot states in Figure 2I and the emissivity at cold/hot states in Figure 2J between porous samples of spacer thickness 150 nm (S1–S6) and 75 nm (S11–S16) are compared, and the values are plotted over the annealing time. Similarly, the *T*_lum(avg)_, *T*_sol_ at cold/hot states (left *y*-axis) and the emissivity at cold/hot states (right *y*-axis) between dense samples of spacer thickness 150 nm (P1–P6) and 75 nm (P11–P16) are compared and the values are plotted versus the annealing time in Figure S3B.

A decrease in *T*_lum(avg)_ and *T*_sol_ at cold and hot states are noticed in porous VO_2_ samples when increasing the spacer thickness from 75 (samples S12–S16) to 150 nm (samples S2–S6) in Figure 2I. A similar trend is also noticed in the dense VO_2_ samples with increasing spacer thickness from 75 nm (samples P12–P16) to 150 nm (samples P2–P6) in Figure S3B. The *T*_lum(avg)_ of sample S0 corresponding to porous VO_2_(35 nm)/ITO/Glass without dielectric spacer is given in Table S2 that has an enhanced *T*_lum(avg)_ of 50.2 % as compared to the porous and dense VO_2_ samples with the dielectric spacer. Also, the *T*_lum(avg)_ of S0 is ∼22 % higher than our previously reported value [29] because of huge decrease in VO_2_ thickness (∼3.7 times) in the present work. The decrease in *T*_lum(avg)_ and *T*_sol_ at cold and hot states in both porous and dense VO_2_ samples are ascribed to the absorption in the visible region due to the increased ZnSe spacer thickness.

Generally, the resonance wavelength, bandwidth, and intensity of absorption bands can be controlled by dielectric spacer layer thickness [22], [49]–[51]. In Figure 2J, the emissivity at the cold state of porous VO_2_ S2–S6 and S12–16 is always lower in the range of 0.1–0.2 irrespective of the spacer thickness as compared to the dense VO_2_ values (Figure S3B). In porous VO_2_ samples with 150 nm ZnSe (samples S2–S6), the Δ*ɛ*_LWIR_ value reaches a maximum of ≥ 0.4 in the samples S4 and S5 annealed at 450 °C for 50 and 60 s (Table 1). Likewise, in porous VO_2_ with 75 nm ZnSe thickness (samples S12–S16), a maximum Δ*ɛ*_LWIR_ of 0.28 is attained in the sample S15 annealed at 450 °C for 60 s (Table S1). The higher emissivity contrast in these porous samples is obtained through the lower emissivity at the cold state and increased emissivity at the hot state. In particular, the *ɛ*_LWIR(hot)_ of S2–S6 are comparatively higher than the S12–S16 (Figure 2J) which can be due to the enhanced absorption in the F–P nanocavity at the metallic R-phase of VO_2_ with increasing spacer thickness. A similar trend is observed in dense VO_2_ samples when the spacer thickness is increased from 75 to 150 nm (Figure S3B). The enhancement in *ɛ*_LWIR(hot)_ and the corresponding increase in Δ*ɛ*_LWIR_ in both porous and dense samples processed for longer annealing times such as 50 and 60 s can be associated with the better crystallinity of VO_2_. Therefore, the emissivity contrast of porous VO_2_ is seen to improve with the increase in the spacer thickness from 75 to 150 nm by sacrificing *T*_lum(avg)_ and *T*_sol_ at cold and hot states.

Among all samples, the VO_2_ sample with porous microstructure annealed at 450 °C for 50 and 60 s (samples S4 and S5) delivered the better emissivity contrast with lower *ɛ*_LWIR_ and higher *T*_sol_ at cold and hot states preserving a good visible transparency. The comparison of emissivity between our work and the values reported in the literature on studies performed in VO_2_-based thin film systems is summarized in Table S3.

**Figure 3:**
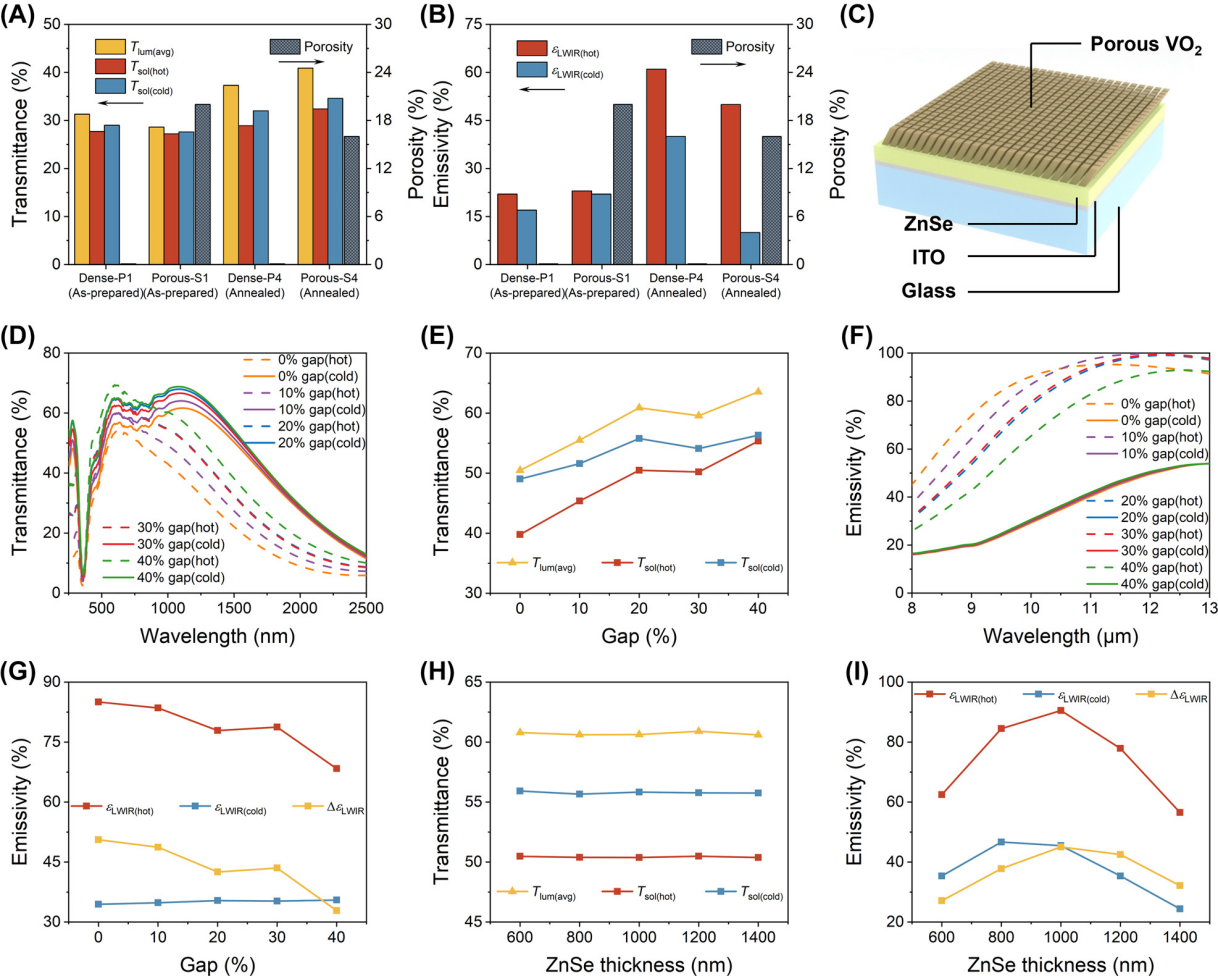
Transmittance and emissivity simulation results on the effect of VO_2_ microstructure variation. (A) Comparison of *T*_lum(avg)_, *T*_sol_ at cold/hot states and estimated porosity between the four samples. (B) Comparison of *ɛ*_LWIR_ at cold/hot states and estimated porosity between the four samples. (C) Schematic diagram of the F–P model containing porous VO_2_ structure for FDTD simulation. (D) Comparison of simulation transmittance spectra between VO_2_ with different gaps in the Vis-NIR range at cold and hot states. (E) *T*_lum(avg)_, *T*_sol_ at cold/hot states versus gaps between VO_2_ columns. (F) Comparison of simulation emissivity spectra between VO_2_ with different gaps in the LWIR range at cold and hot states. (G) *ɛ*_LWIR_ at cold/hot states and Δ*ɛ*_LWIR_ versus gaps between VO_2_ columns. (H) *T*_lum(avg)_, *T*_sol_ at cold/hot states versus thickness of ZnSe spacer. (I) *ɛ*_LWIR_ at cold/hot states and Δ*ɛ*_LWIR_ versus thickness of ZnSe spacer.

In Figure 3A and B, the process-microstructure-property relationship is compared between the samples of porous (S1-as-prepared, S4-annealed) and dense (P1-as-prepared, P4-annealed) microstructure. The porosity percentages are estimated from SEM analysis. Both as-prepared porous and dense V samples S1 and P1 have lower emissivity at both cold and hot states, with nearly zero emissivity contrast and inadequate *T*_lum(avg)_. The annealed dense VO_2_ sample P4 shows higher emissivity at both cold (0.4) and hot (0.61) states with *T*_lum(avg)_ of 37.3 % lower than the optimal. Whereas the porous VO_2_ sample S4 annealed at 450 °C for 50 s has lower emissivity at cold (0.1) and higher emissivity at hot (0.5) state, and an enhanced *T*_lum(avg)_ of 40.9 % along with a higher *T*_sol_. The result suggests that the performance of porous VO_2_ sample S4 is better as compared to dense samples for emissivity regulation of a thermochromic window in colder regions.

### 3.3 FDTD simulation

FDTD simulation is conducted to further explore the effects of VO_2_ porosity on related performance. Figure 3C depicts the simulation model with a porous VO_2_ structure. It should be noted that this is an idealized model and the actual case is a bit more complex. At first, the thickness of ZnSe and ITO is set to 1200 nm and 200 nm, respectively. The different porosities are achieved by adjusting the gap between VO_2_ tilting columns of the same size. It is found that emissivity regulation is affected by both VO_2_ thickness and porosity (Figure S4). Here, the VO_2_ thickness is fixed at 30 nm for subsequent studies. Figure 3D shows the comparison of simulated transmittance spectra in Vis-NIR range between F–P cavity systems containing different porous VO_2_. Transmittance differences between hot and cold states occur mainly in the NIR region, which is consistent with experimental results. We can also find that the porosity of VO_2_ has a greater effect on transmittance in high temperatures than in low temperatures. The calculated *T*_lum(avg)_ and *T*_sol_ in cold/hot states with different porosities are summarized in Figure 3E. It can be concluded that both *T*_lum(avg)_ and *T*_sol_ tend to increase with increasing gaps. However, the difference in *T*_sol_ between the hot and cold states is getting closer, which means weakened solar modulation because of the diminished mass of VO_2_. When the gap reaches 40 %, it almost no longer has solar modulation capability. Figure 3F shows the simulated emissivity spectra in LWIR. The porosity of VO_2_ greatly influences the emission in high temperatures whereas the spectra in low temperatures are almost identical. *ɛ*_LWIR(hot)_, *ɛ*_LWIR(cold)_, and Δ*ɛ*_LWIR_ with different porosities are calculated to verify this point (Figure 3G). There is a decreasing trend for *ɛ*_LWIR(hot)_ with increased VO_2_ porosity, while *ɛ*_LWIR(cold)_ is at a relatively stable value below 0.4. We suppose the *ɛ*_LWIR(cold)_ discrepancy between experiment and simulation can be ascribed to that the *n*, and *k* values taken from previous reports could vary from the real samples, and the potential impurities, crystallinity, and morphology non uniformity in the real samples.

We also attempt to use the actual thickness in experiment (150 nm-thick ZnSe) as the simulation parameter (Figure S5). The results show the same trend as the case of 1200 nm-thick ZnSe. But the values concerning *ɛ*_LWIR_ are on a low order of magnitude, which can also be attributed to the aforementioned factors. Reasonable porous structure construction is a key point that can offer an enhanced *T*_lum(avg)_ and depressed *ɛ*_LWIR(hot)_ without weakening solar modulation and cooling regulation too much. In this thickness design, a 20 % gap is a reasonable structure for application in the cold region as its *T*_lum(avg)_ is up to 60.9 % with the Δ*T*_sol_ of 5.3 %, and *ɛ*_LWIR_ in hot and cold states are 0.78 and 0.35 by considering its relatively high *T*_lum(avg)_, *T*_sol(cold)_, and low *ɛ*_LWIR(cold)_. Besides, the relationships between performance and the thickness of ZnSe are simulated, as shown in Figure 3H and I. The corresponding spectra are shown in Figure S6. The simulation results reveal that the thickness variation of the ZnSe spacer we selected has a smaller effect on the transmittance in Vis-NIR. But there is a great difference in *ɛ*_LWIR_. As the ZnSe thickness increases, both *ɛ*_LWIR(hot)_ and *ɛ*_LWIR(cold)_ increase and then decrease, thus leading to a similar trend in Δ*ɛ*_LWIR_. It reveals that there is an optimized spacer thickness for the best emissivity switching performance, which agrees with the study on VO_2_/PMMA/ITO/Glass/ITO RCRT smart window [4].

### 3.4 EnergyPlus simulation

**Figure 4:**
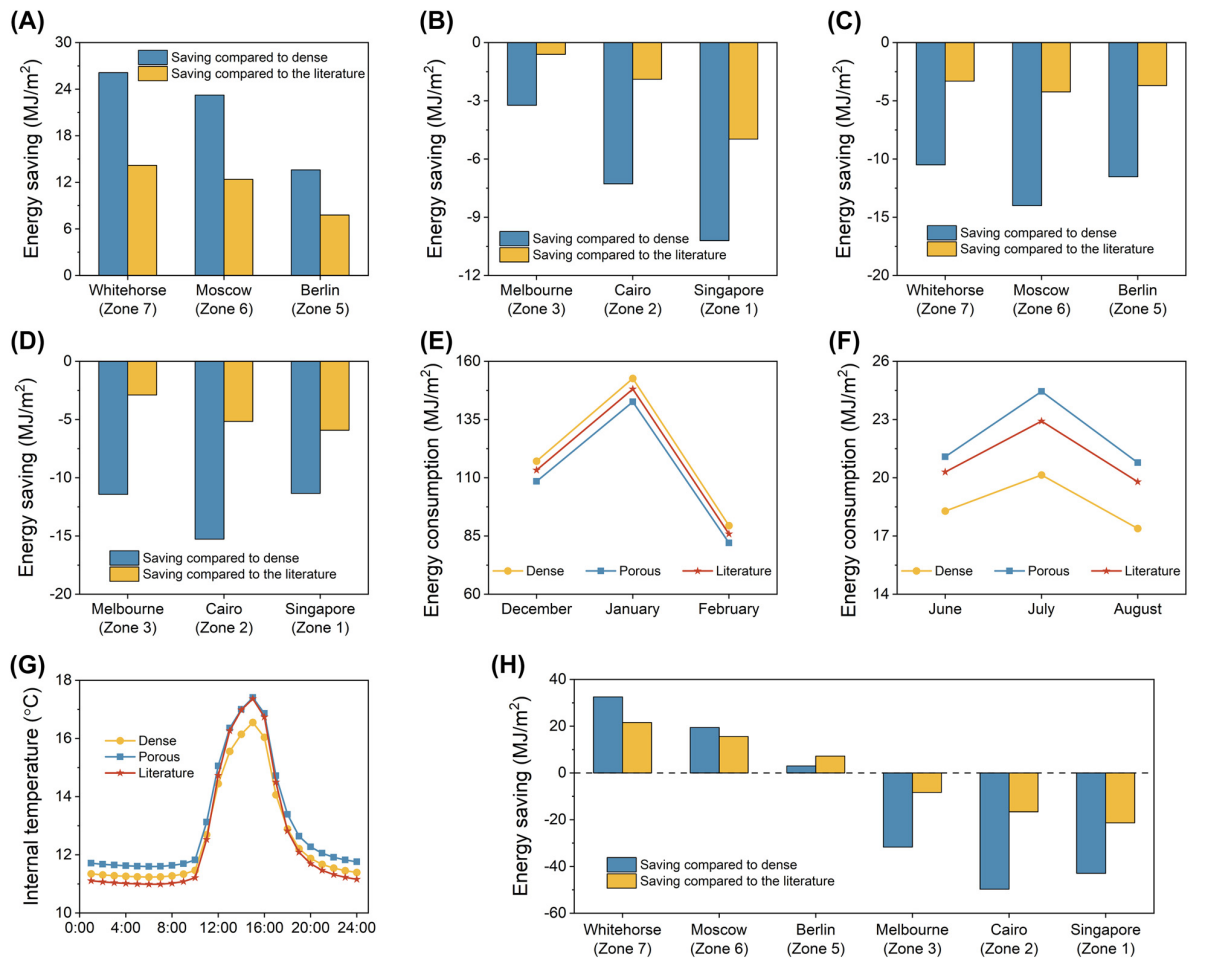
Energy consumption in different cities applying porous and dense VO_2_. Winter energy saving of porous VO_2_ compared to dense VO_2_ and the literature in (A) cold regions and (B) hot regions. Summer energy saving of porous VO_2_ compared to dense VO_2_ and the literature in (C) cold regions and (D) hot regions. Detailed monthly energy consumption with the three samples in Whitehorse in (E) winter and (F) summer. (G) The all-day internal temperature with three samples in Whitehorse on January 21th. (H) Annual energy saving of porous VO_2_ compared to dense VO_2_ and the literature in the six cities.

As the porous VO_2_ structure has a tuneable *ɛ*_LWIR(cold)_ with the range of 0.1–0.3, this film is suitable for cold climate regions where low emissivity is necessary [4], [52]. The energy-saving performance of thermochromic porous VO_2_ structure is investigated through actual building energy consumption simulation compared with dense VO_2_ and the RCRT window we previously proposed [4]. The weather data of Whitehorse, Moscow, Berlin, Melbourne, Cairo, and Singapore are used in this simulation [36]. In the classification of ASHARE, they belong to cold climate Zone 7, 6, 5, 3, 2, and 1, respectively. Among them, Whitehorse, Moscow, and Berlin are in relatively cold climate zones, where heating needs are greater. Figure 4A and B shows the winter energy-saving performance of porous VO_2_ compared to dense VO_2_ and the literature in different cities, which is calculated by using the energy consumption of the dense VO_2_/literature minus that of porous VO_2_. It should be pointed out that except for Melbourne, the winter months are December, January, and February while June, July, and August are winter months in Melbourne. For the summer months, they are the three months counted from the fourth month after the end of winter. It can be seen that porous VO_2_ does have the advantage of low winter energy consumption in cold regions whereas its winter energy consumption is higher in hot regions. Regarding summer energy-saving performance, porous VO_2_ shows a negative effect in both cold and hot regions (Figure 4C and D).

Nevertheless, the negative summer saving in cold regions is much smaller than the positive winter saving. Take Whitehorse as an example, the winter energy consumption is much more than summer due to huge heating demand, as shown in Figure 4E and F. During the winter months, there can be savings close to 10 MJ/m^2^ per month compared to dense VO_2_, while the negative saving is just 1−3 MJ/m^2^ per month in summer. Detailed energy consumption in winter and summer of other cities is presented in Figures S7 and 8. Also, we simulate the winter/summer internal temperature of the six cities. Figure 4G illustrates the winter internal temperature in Whitehorse. Compared to other data in Figures S9 and 10, it can be concluded that porous VO_2_ offer a more comfortable winter internal temperature than dense VO_2_ and the literature in cold regions. The annual energy saving of porous VO_2_ in the six cities is shown in Figure 4H. Due to superior saving performance in winter for cold regions, porous VO_2_ exhibits an overall saving effect compared to dense VO_2_ and the literature, whereas it is inferior to them in hot regions, which shows a customized design for particular cities. The simulation results demonstrate that the thermochromic porous sample shows promising energy-saving performance in multiple cities where heating demand is predominant.

## 4 Conclusions

The VO_2_/ZnSe/ITO/Glass Fabry−Perot (F–P) cavity systems with porous and dense VO_2_ microstructure are successfully fabricated through industrially desirable sputtering and thermal evaporation methods. The Vis-NIR transmittance and emissivity spectra of porous and dense VO_2_ demonstrate enhanced visible transparency and improved emissivity switching in systems with porous VO_2_. An enhancement in emissivity contrast Δ*ɛ*_LWIR_ of 0.4 (*ɛ*_LWIR(cold)_ = 0.1 and *ɛ*_LWIR(hot)_ = 0.5) and 0.42 (*ɛ*_LWIR(cold)_ = 0.11 and *ɛ*_LWIR(hot)_ = 0.53) is obtained in porous VO_2_(35 nm)/ZnSe(150 nm) samples annealed for 50 and 60 s, respectively, retaining *T*_lum_ between 40 and 42 % in cold and hot states. The low emissivity value at a cold state is appropriate to apply in cold climate regions. In FDTD simulation, an emissivity contrast of 0.43 (*ɛ*_LWIR(cold)_ = 0.35 and *ɛ*_LWIR(hot)_ = 0.78) with an enhanced *T*_lum(avg)_ of 60.9 % is achieved in the F–P cavity that contains 30 nm-thick porous VO_2_ with 20 % gap and 1200 nm-thick ZnSe. Both the experimental *T*_lum_ value of porous VO_2_ annealed at 450 °C for 50 and 60 s and simulated *T*_lum_ values are higher than the dense VO_2_ which is indispensable for the visual comfort of the building’s interior. Furthermore, the low *ɛ*_LWIR_ at low temperatures provides a customized strategy for specific cities with high heating demand. By comparing the simulated energy consumption of F–P systems based on porous VO_2_, dense VO_2,_ and the RCRT window previously reported in six cities of different climate zones, it can be concluded that porous VO_2_ has a superior saving performance in winter for cold regions.

## Supplementary Material

Supplementary Material Details
