# Supplementary material for: Porous vanadium dioxide thin film-based Fabry−Perot cavity system for radiative cooling regulating thermochromic windows: experimental and simulation studies
Source: Nanophotonics. 2024 Jan 18;13(5):711–23. doi: 10.1515/nanoph-2023-0716 (PMC11501869; doi:10.1515/nanoph-2023-0716)
Supplement: Supplementary file 1 — Supplementary Material Details [file j_nanoph-2023-0716_suppl_001.docx]

**Supporting Information**

**Porous Vanadium Dioxide Thin Film-based Fabry−Perot Cavity System for Radiative Cooling Regulating Thermochromic Windows: Experimental and Simulation Studies**

*Saranya Bhupathi, Shancheng Wang, Guanya Wang, and Yi Long**

***Corresponding author:** **Yi Long,** Department of Electronic Engineering, The Chinese University of Hong Kong, Shatin, New Territories, Hong Kong SAR 999077, China; yilong@cuhk.edu.hk; https://orcid.org/0000-0003-0608-8353

**Saranya Bhupathi,** School of Materials Science and Engineering, Nanyang Technological University, 50 Nanyang Avenue, 639798, Singapore, and Singapore-HUJ Alliance for Research and Enterprise (SHARE), Campus for Research Excellence and Technological Enterprise (CREATE), 138602, Singapore; amsaranya.phy@gmail.com; https://orcid.org/0000-0001-9367-778X

**Shancheng Wang,** **Guanya Wang,** Department of Electronic Engineering, The Chinese University of Hong Kong, Shatin, New Territories, Hong Kong SAR 999077, China; shanchengwang@cuhk.edu.hk (S. Wang); guanyawang@link.cuhk.edu.hk (G. Wang); https://orcid.org/0000-0001-5817-2217 (S. Wang); https://orcid.org/0000-0002-7813-0230 (G.Wang)

**Supplementary note 1: Apartment model in building energy consumption simulation.**


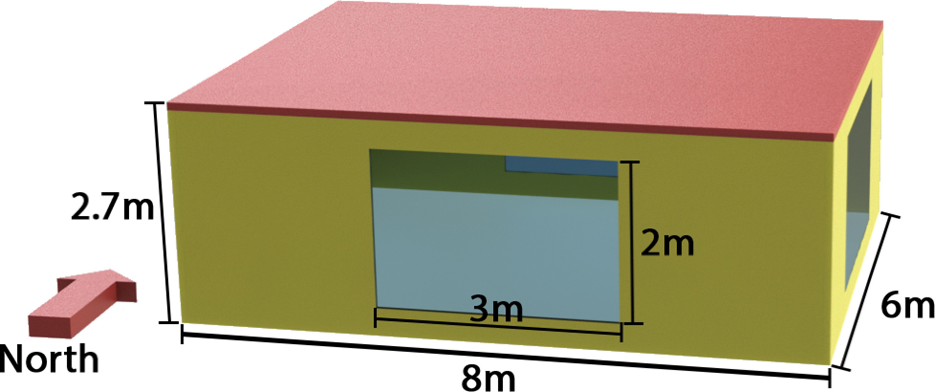


**Figure S1. Apartment model in building energy consumption simulation:** Figure of apartment model used in the building energy consumption simulation with the dimension of 8 m length, 6 m width, and 2.7 m height.

**Supplementary note 2: AFM images of porous and dense VO_2_.**


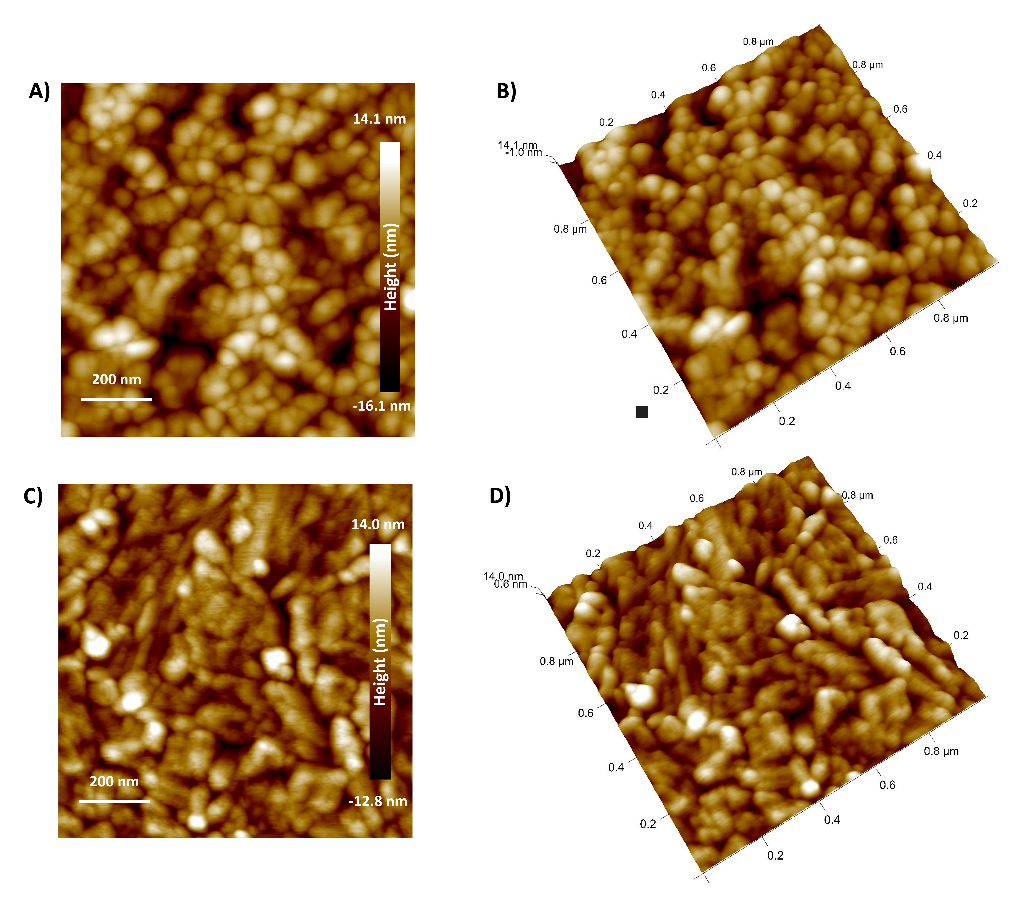


**Figure S2. AFM images of porous and dense VO_2_:** AFM (A) 2D and (B) 3D images of porous VO_2_ sample S4. AFM (C) 2D and (D) 3D images of dense VO_2_ sample P4. S4 corresponds to porous VO_2_(35 nm)/ZnSe(150 nm) and P4 corresponds to dense VO_2_(35 nm)/ZnSe(150 nm). Both samples S4 and P4 are annealed at 450 °C for 50 sec in the ambient air.

**Table S1:** Summary of experimental thermochromic and emissivity parameters of porous and dense V and VO_2_ samples with sample codes S11 to S16 and P11 to P16. The annealing temperature of samples S12 to S16 and P12 to P16 is 450 °C.

| **Code** | **Layer and thickness details** | **Annealing time (sec)** | $\boldsymbol{T}_{\mathbf{lum}}$ **(%)** | | $\boldsymbol{T}_{\mathbf{lum}\mathbf{(}\mathbf{avg}\mathbf{)}}$  **(%)** | ${\boldsymbol{\Delta}\boldsymbol{T}}_{\mathbf{NIR}}$  **(%)** | $\boldsymbol{T}_{\mathbf{sol}}$ **(%)** | | ${\boldsymbol{\Delta}\boldsymbol{T}}_{\mathbf{sol}}$ **(%)** | $\boldsymbol{\varepsilon}_{\mathbf{LWIR}}$ | | ${\boldsymbol{\Delta}\boldsymbol{\varepsilon}}_{\mathbf{LWIR}}$ |
| --- | --- | --- | --- | --- | --- | --- | --- | --- | --- | --- | --- | --- |
|  |  |  | **20 °C** | **100 °C** |  |  | **20 °C** | **100 °C** |  | **20 °C** | **100 °C** |  |
| S11 | Porous V(35 nm)/ZnSe(75 nm) | As-prepared | 35.3 | 34.5 | 34.9 | 0.0 | 31.8 | 31.3 | 0.5 | 0.21 | 0.25 | 0.04 |
| S12 | Porous VO_2_(35 nm)/ZnSe(75 nm) | 30 | 42.7 | 41.7 | 42.2 | 1.4 | 35.8 | 33.8 | 2.1 | 0.22 | 0.30 | 0.08 |
| S13 | Porous VO_2_(35 nm)/ZnSe(75 nm) | 40 | 43.5 | 42.4 | 43.0 | 1.7 | 35.7 | 33.6 | 2.2 | 0.22 | 0.33 | 0.11 |
| S14 | Porous VO_2_(35 nm)/ZnSe(75 nm) | 50 | 43.7 | 42.0 | 42.9 | 0.3 | 35.3 | 34.3 | 1.0 | 0.11 | 0.34 | 0.23 |
| S15 | Porous VO_2_(35 nm)/ZnSe(75 nm) | 60 | 47.7 | 46.5 | 47.1 | 0.2 | 37.8 | 37.0 | 0.8 | 0.12 | 0.40 | 0.28 |
| S16 | Porous VO_2_(35 nm)/ZnSe(75 nm) | 70 | 45.3 | 44.2 | 44.8 | 0.9 | 36.9 | 35.5 | 1.4 | 0.10 | 0.34 | 0.24 |
| P11 | Dense V(35 nm)/ZnSe(75 nm) | As-prepared | 34.3 | 33.1 | 33.7 | 0.4 | 30.0 | 29.0 | 1.1 | 0.14 | 0.14 | 0.00 |
| P12 | Dense VO_2_(35 nm)/ZnSe(75 nm) | 30 | 40.6 | 40.1 | 40.4 | 2.5 | 33.5 | 30.3 | 3.2 | 0.24 | 0.42 | 0.18 |
| P13 | Dense VO_2_(35 nm)/ZnSe(75 nm) | 40 | 41.5 | 41.6 | 41.6 | 2.7 | 35.9 | 32.6 | 3.2 | 0.24 | 0.45 | 0.21 |
| P14 | Dense VO_2_(35 nm)/ZnSe(75 nm) | 50 | 39.4 | 39.2 | 39.3 | 2.8 | 33.2 | 29.8 | 3.3 | 0.26 | 0.48 | 0.22 |
| P15 | Dense VO_2_(35 nm)/ZnSe(75 nm) | 60 | 38.9 | 39.5 | 39.2 | 2.4 | 33.0 | 30.3 | 2.7 | 0.28 | 0.47 | 0.19 |
| P16 | Dense VO_2_(35 nm)/ZnSe(75 nm) | 70 | 40.3 | 41.0 | 40.7 | 2.3 | 34.0 | 31.5 | 2.5 | 0.28 | 0.46 | 0.18 |

**Supplementary note 3: Microstructure and spacer thickness effect on the thermochromic and emissivity parameters**


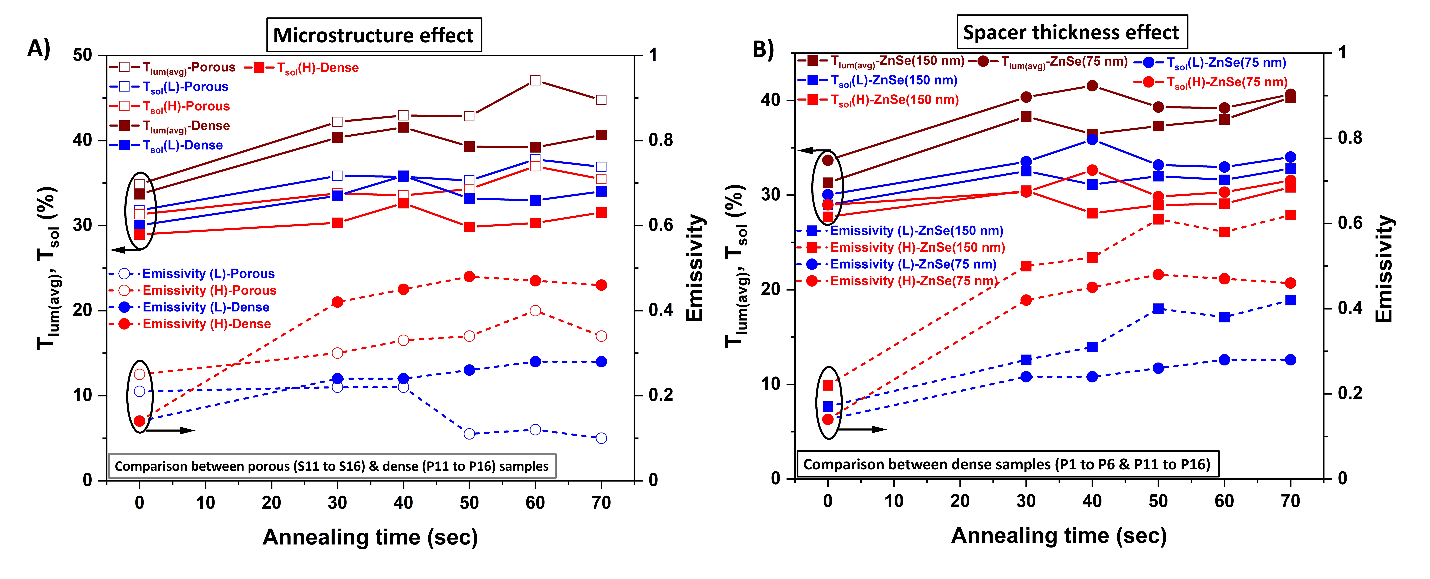


**Figure S3. Microstructure and spacer thickness effect on the thermochromic and emissivity parameters:** (A) Comparison of $T_{lum(avg)}$, $T_{sol}$ at cold/hot states (left y-axis), and emissivity at cold/hot states (right y-axis) between samples of porous (S11 to S16) and dense (P11 to P16) microstructure versus annealing time. (B) Comparison of $T_{lum(avg)}$, $T_{sol}$ at cold/hot states (left y-axis), and emissivity at cold/hot states (right y-axis) between dense samples of different spacer thicknesses such as 150 nm (P1 to P6) and 75 nm (P11 to P16) versus annealing time. L represents cold state which are represented in blue lines. H represents hot state which are represented in red lines. Sample S11 corresponds to as-prepared porous V(35 nm)/ZnSe(75 nm) without heat treatment. Samples S12 to S16 correspond to porous VO_2_(35 nm)/ZnSe(75 nm), heat treated at 450 °C for 30, 40, 50, 60, and 70 sec, respectively. Sample P11 corresponds to as-prepared dense V(35 nm)/ZnSe(75 nm) without heat treatment. Samples P12 to P16 correspond to dense VO_2_(35 nm)/ZnSe(75 nm) heat treated at 450 °C for 30, 40, 50, 60, and 70 sec, respectively. Sample P1 corresponds to as-prepared dense V(35 nm)/ZnSe(150 nm) without heat treatment. Samples P2 to P6 correspond to dense VO_2_(35 nm)/ZnSe(150 nm) heat treated at 450 °C for 30, 40, 50, 60, and 70 sec, respectively.

**Table S2:** Summary of experimental thermochromic and emissivity parameters of samples T1 and S0. T1 corresponds to ITO-coated glass substrate and S0 corresponds to porous VO_2_(35 nm)/ITO/Glass without ZnSe spacer, heat treated at 450 °C for 50 sec.

| **Sample code** | **Sample details with annealing temperature and time** | $\boldsymbol{T}_{\mathbf{lum}}$ **(%)** | | $\boldsymbol{T}_{\mathbf{lum}\mathbf{(}\mathbf{avg}\mathbf{)}}$  **(%)** | ${\boldsymbol{\Delta}\boldsymbol{T}}_{\mathbf{NIR}}$  **(%)** | $\boldsymbol{T}_{\mathbf{sol}}$ **(%)** | | ${\boldsymbol{\Delta}\boldsymbol{T}}_{\mathbf{sol}}$ **(%)** | $\boldsymbol{\varepsilon}_{\mathbf{LWIR}}$ | | ${\boldsymbol{\Delta}\boldsymbol{\varepsilon}}_{\mathbf{LWIR}}$ |
| --- | --- | --- | --- | --- | --- | --- | --- | --- | --- | --- | --- |
|  |  | **20 °C** | **100 °C** |  |  | **20 °C** | **100 °C** |  | **20 °C** | **100 °C** |  |
| T1 | ITO-coated Glass | 84.1 | 83.3 | 83.7 | 0.2 | 76.0 | 75.3 | 0.8 | 0.14 | 0.17 | 0.03 |
| S0 | Porous VO_2_/ITO/G (450 °C/50 sec) | 49.3 | 51.0 | 50.2 | 3.2 | 41.1 | 38.8 | 2.3 | 0.12 | 0.37 | 0.25 |

**Table S3:** Comparison of emissivity values between present work and literature.

| **VO_2_** | **Sample structure** | **Fabrication method** | **Thermal management** | $\boldsymbol{T}_{\mathbf{vis}}$ **(%)** | | $\boldsymbol{\varepsilon}_{\mathbf{LWIR}}$ | | ${\boldsymbol{\Delta}\boldsymbol{\varepsilon}}_{\mathbf{LWIR}}$ |
| --- | --- | --- | --- | --- | --- | --- | --- | --- |
|  |  |  |  | **Cold** | **Hot** | **Cold** | **Hot** |  |
| Planar | VO_2_/HfO_2_/Al[1] | High-power impulse magnetron sputtering, DC Sputtering | Buildings, vehicles |  |  | 0.21 | 0.82 | 0.61 |
| Planar | VO_2_/BaF_2_/Gold with Si layer on top[2] | Pulsed laser deposition, e-beam evaporation | Spacecraft |  |  | 0.15 | 0.64 | 0.49 |
| Planar | VO_2_/HfO_2_/Ag/Si[3] | Reactive sputtering, e-beam evaporation, DC sputtering |  |  |  | 0.13 | 0.68 | 0.55 |
| Planar | VO_2_/SiO_2_/Au/Quartz[4] | Reactive pulsed laser deposition, plasma  enhanced chemical  vapor deposition, RF sputtering | Spacecraft |  |  | 0.8 | 0.32 | 0.48 |
| Planar | VO_2_/PMMA/ITO/Glass/ITO[5] (**previous work**) | Spin coating | Buildings | 27.8 | 26.1 | 0.61 | 0.21 | 0.4 |
| Metasurface | VO_2_/SiO_2_/Al/Al_2_O_3_/Si with Al_2_O_3_ capping layer[6] | Sputtering, plasma  enhanced chemical  vapor deposition | Spacecraft |  |  |  |  | 0.48 |
| Metasurface | VO_2_/SiO_2_/AZO/CaF_2_[7] | Atomic layer deposition, plasma  enhanced chemical  vapor deposition, e-beam lithography, ion beam etching | Space and terrestrial application | 55 | 58 | 0.54 | 0.81 | 0.26 |
| Microsphere | CaF_2_/VO_2_ Core–Shell structure[8] | Solvent/hydrothermal-calcination |  |  |  | 0.47 | 0.83 | 0.36 |
| Porous and tilted | VO_2_/ZnSe/ITO/Glass (**This work**) | RF sputtering, thermal evaporation | Buildings | 41.8 | 40.0 | 0.1 | 0.5 | 0.4 |

**Supplementary note 4: FDTD simulation results of emissivity in LWIR with different VO_2_ thicknesses and porosities**


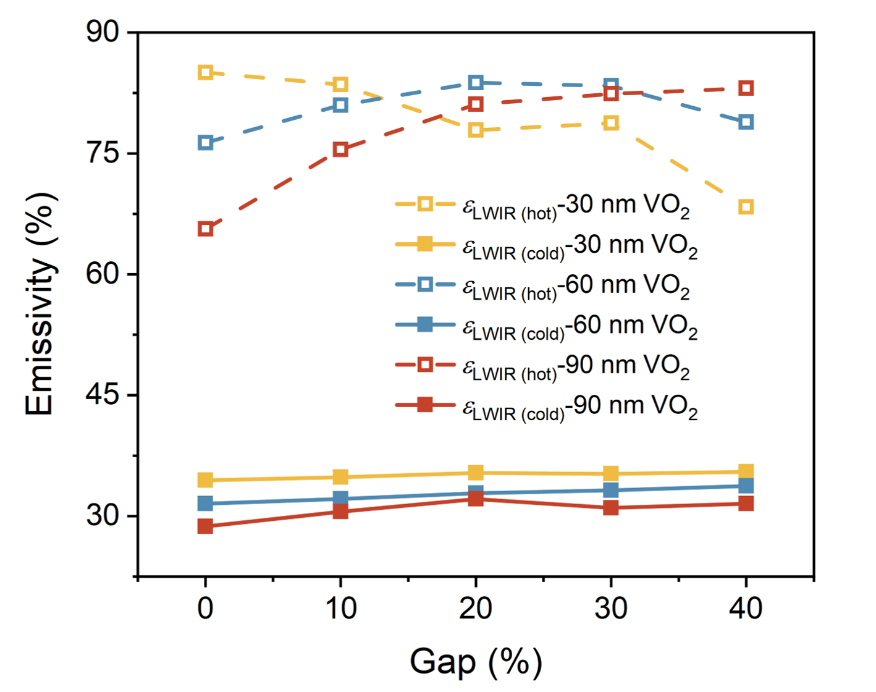


**Figure S4:** $\varepsilon_{LWIR}$ at cold/hot states and ${\Delta\varepsilon}_{LWIR}$ versus gaps between VO_2_ columns with 30, 60, and 90 nm thickness.

**Supplementary note 5: FDTD simulation results of emissivity in LWIR with different VO_2_ thicknesses and porosities**


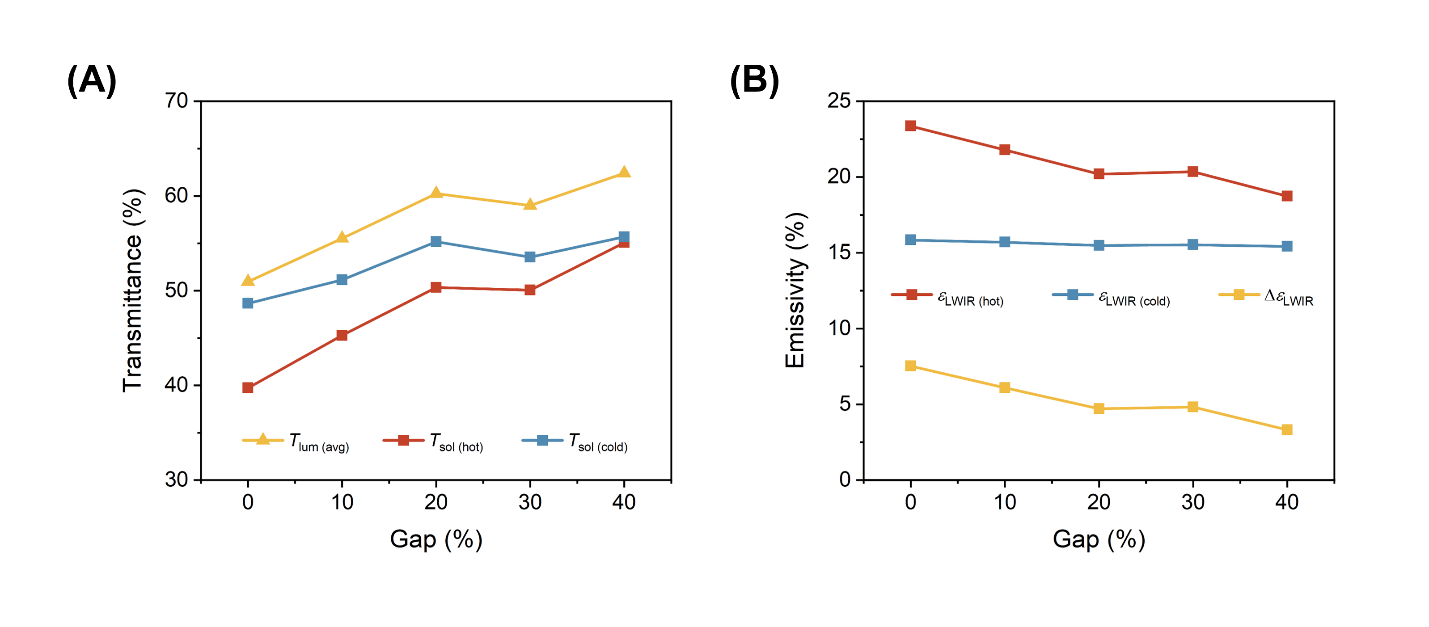


**Figure S5:** $\varepsilon_{LWIR}$ at cold/hot states and ${\Delta\varepsilon}_{LWIR}$ versus gaps between VO_2_ columns with 30, 60, and 90 nm thickness.

**Supplementary note 6: FDTD simulation spectra of F-P cavities with different ZnSe thicknesses**

**
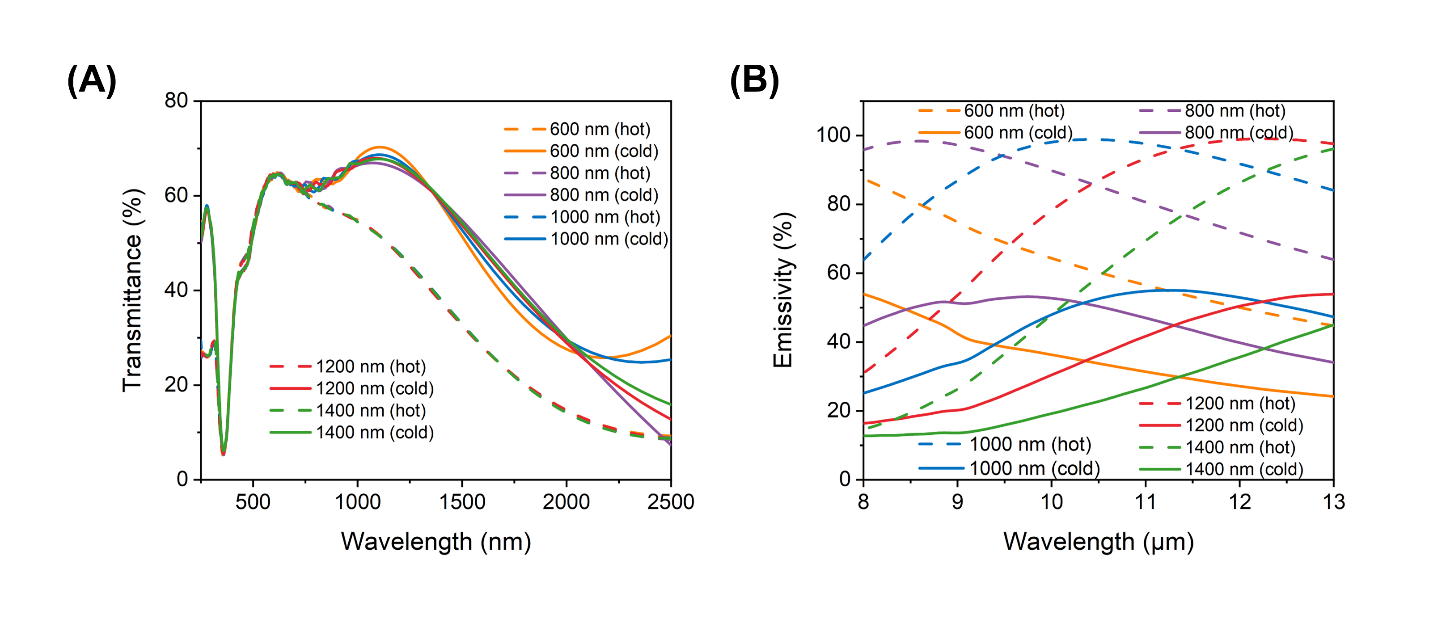
**

**Figure S6:** (A) Comparison of simulation transmittance spectra between ZnSe spacer with different thicknesses in the Vis-NIR range at cold and hot states. (B) Comparison of simulation emissivity spectra between ZnSe spacer with different thicknesses in the LWIR range at cold and hot states.

**Supplementary note 7: EnergyPlus simulation of winter monthly energy consumption with the three samples in the five cities in Zone 6, 5, 3, 2, 1**


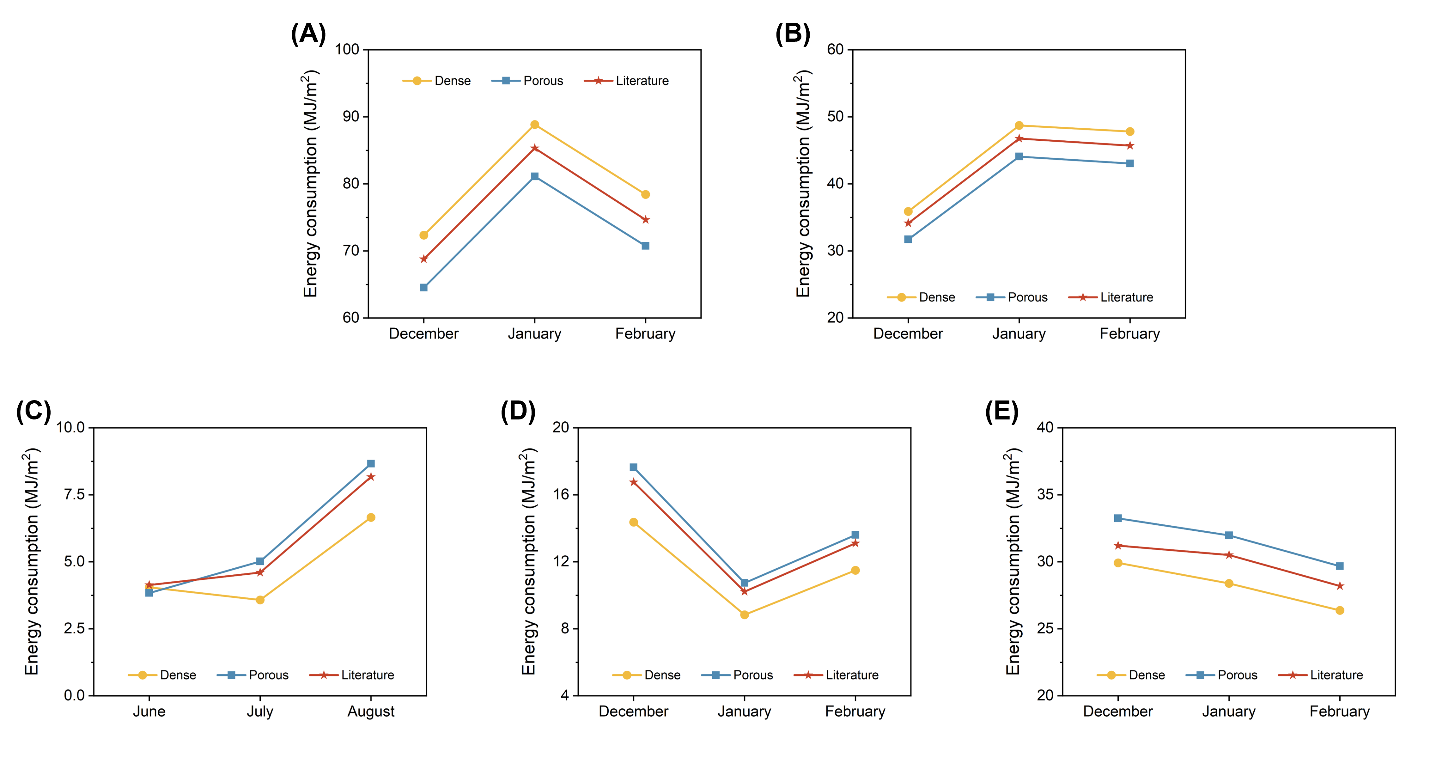


**Figure S7:** Winter monthly energy consumption with the three samples in (A) Moscow, (B) Berlin, (C) Melbourne, (D) Cairo, and (E) Singapore.

**Supplementary note 8: EnergyPlus simulation of summer monthly energy consumption with the three samples in the five cities in Zone 6, 5, 3, 2, 1**


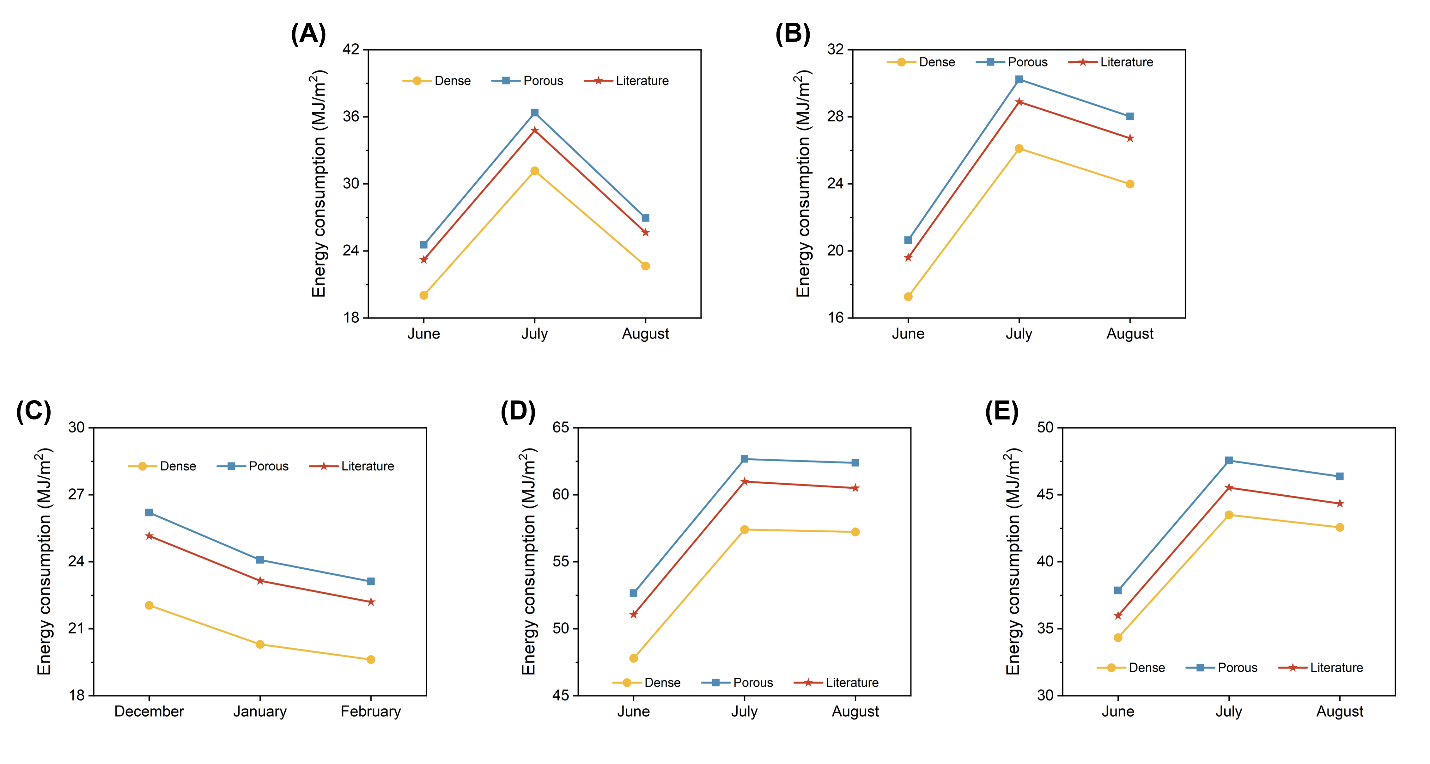


**Figure S8:** Summer monthly energy consumption with the three samples in (A) Moscow, (B) Berlin, (C) Melbourne, (D) Cairo, and (E) Singapore.

**Supplementary note 9: EnergyPlus simulation of winter all-day internal temperature with the three samples in the five cities in Zone 6, 5, 3, 2, 1**


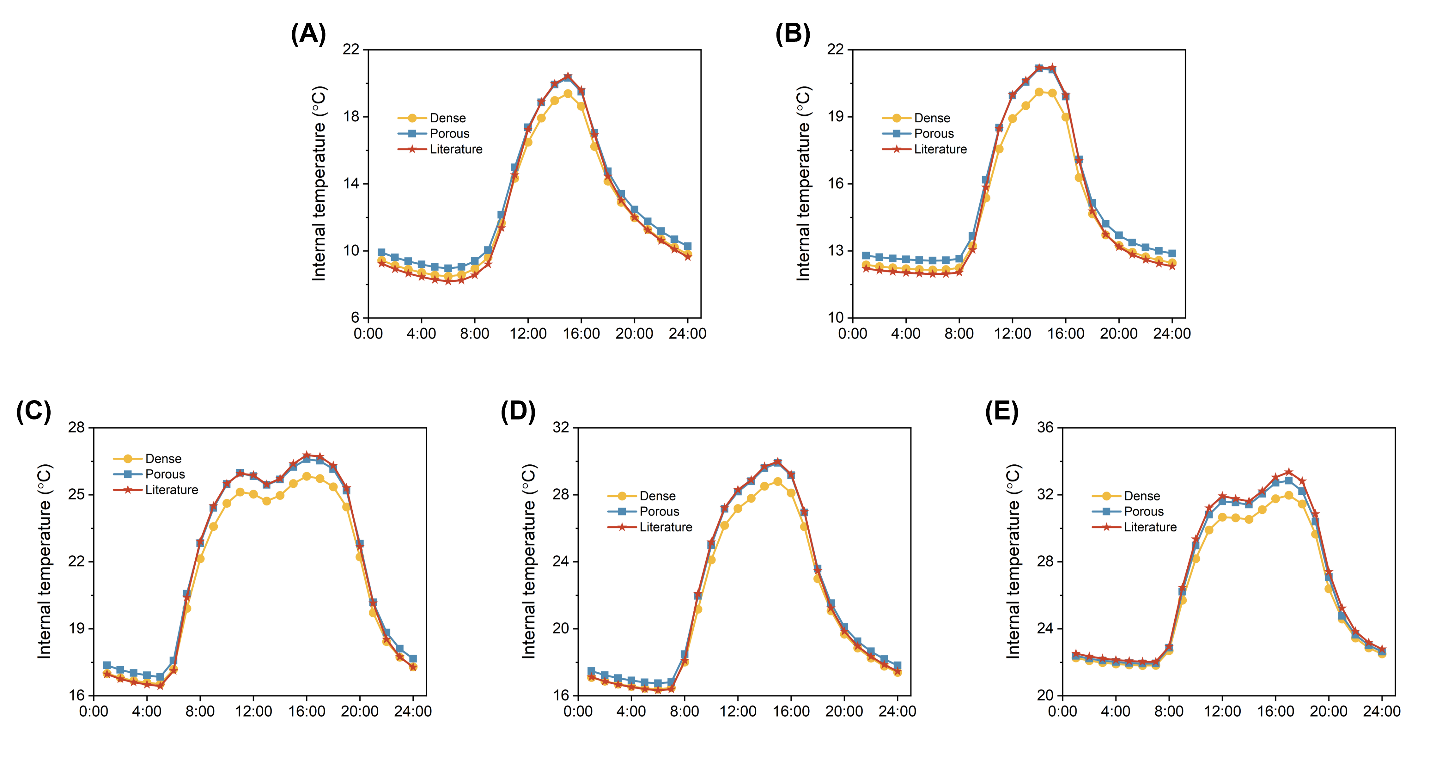


**Figure S9:** Winter all-day internal temperature with the three samples in (A) Moscow, (B) Berlin, (C) Melbourne, (D) Cairo, and (E) Singapore.

**Supplementary note 10: EnergyPlus simulation of summer all-day internal temperature with the three samples in the six cities in Zone 7, 6, 5, 3, 2, 1**

**
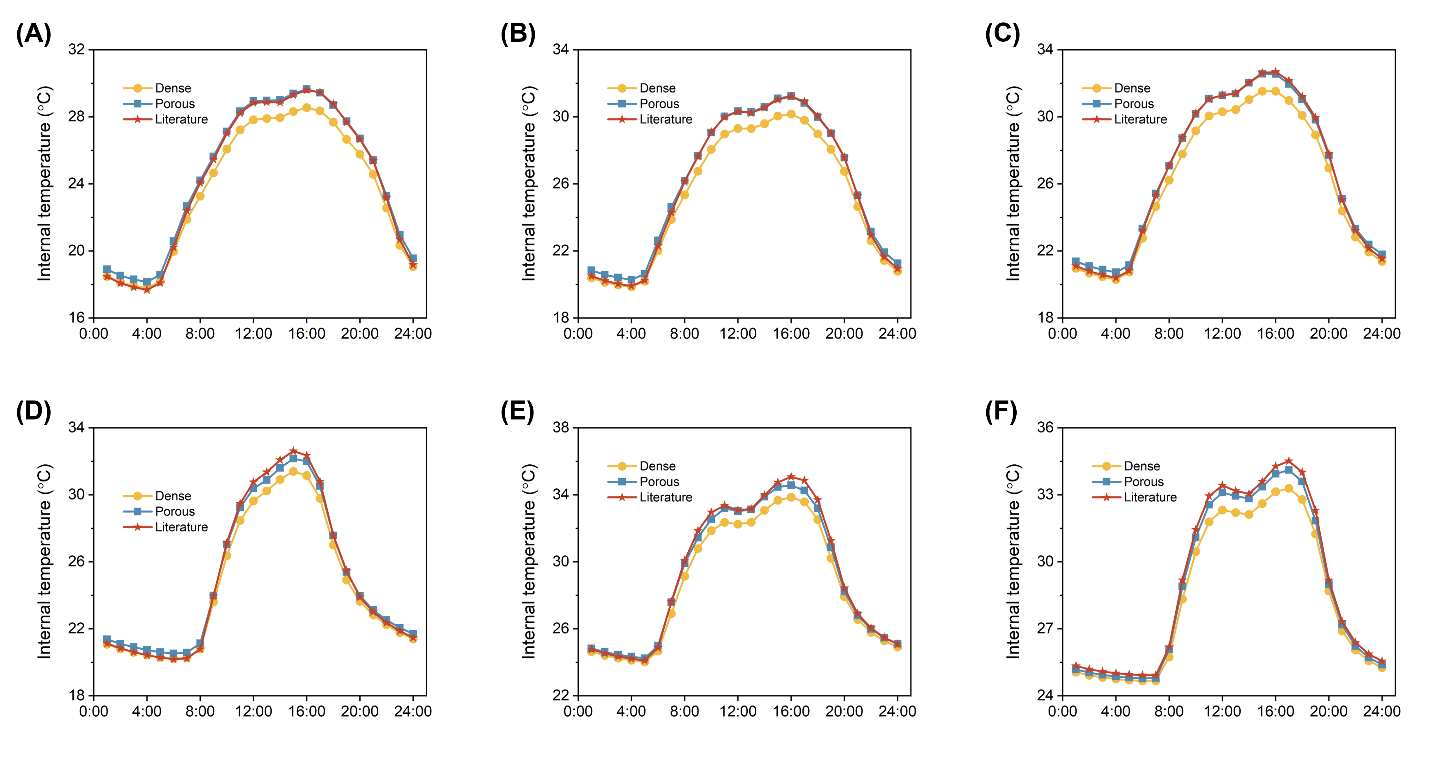
**

**Figure S10:** Summer all-day internal temperature with the three samples in (A) Whitehorse, (B) Moscow, (C) Berlin, (D) Melbourne, (E) Cairo, and (F) Singapore.

**References**

[1] J. Gu et al., “VO_2_-based infrared radiation regulator with excellent dynamic thermal management performance,” *ACS Appl Mater Interfaces*, vol. 14, no. 2, pp. 2683–2690, Jan. 2022, doi: 10.1021/acsami.1c17914.

[2] H. Kim et al., “VO_2_-based switchable radiator for spacecraft thermal control,” *Sci Rep*, vol. 9, no. 1, p. 11329, Aug. 2019, doi: 10.1038/s41598-019-47572-z.

[3] X. Wang, Y. Cao, Y. Zhang, L. Yan, and Y. Li, “Fabrication of VO_2_-based multilayer structure with variable emittance,” *Appl Surf Sci*, vol. 344, pp. 230–235, Jul. 2015, doi: 10.1016/j.apsusc.2015.03.116.

[4] A. Hendaoui, N. Émond, S. Dorval, M. Chaker, and E. Haddad, “VO_2_-based smart coatings with improved emittance-switching properties for an energy-efficient near room-temperature thermal control of spacecrafts,” *Solar Energy Materials and Solar Cells*, vol. 117, pp. 494–498, Oct. 2013, doi: 10.1016/j.solmat.2013.07.023.

[5] S. Wang, T. Jiang, Y. Meng, R. Yang, G. Tan, and Y. Long, “Scalable thermochromic smart windows with passive radiative cooling regulation,” *Science* (1979), vol. 374, no. 6574, pp. 1501–1504, Dec. 2021, doi: 10.1126/science.abg0291.

[6] K. Sun et al., “VO_2_ thermochromic metamaterial-based smart optical solar reflector,” *ACS Photonics*, vol. 5, no. 6, pp. 2280–2286, Jun. 2018, doi: 10.1021/acsphotonics.8b00119.

[7] K. Sun et al., “VO_2_ metasurface smart thermal emitter with high visual transparency for passive radiative cooling regulation in space and terrestrial applications,” *Nanophotonics*, vol. 11, no. 17, pp. 4101–4114, Aug. 2022, doi: 10.1515/nanoph-2022-0020.

[8] X. Wu, L. Yuan, X. Weng, L. Qi, B. Wei, and W. He, “Passive Smart Thermal Control Coatings Incorporating CaF_2_/VO_2_ Core–Shell Microsphere Structures,” *Nano Lett*, vol. 21, no. 9, pp. 3908–3914, May 2021, doi: 10.1021/acs.nanolett.1c00454.
